# Incidence of intensive care unit acquired weakness in critically ill patients treated with kidney replacement therapy: A systematic review and meta-analysis

**DOI:** 10.1371/journal.pone.0323874

**Published:** 2025-05-15

**Authors:** Tao Yang, Kaikai Zhang, Xiuming Xi, Shanshan Yu

**Affiliations:** 1 Department of Critical Care Medicine, Beijing Tiantan Hospital, Capital Medical University, Beijing, China; 2 Department of Emergency Medicine, Jinyang Hospital Affiliated of Guizhou Medical University, Guiyang Second People’s Hospital, Guiyang, China; 3 Department of Critical Care Medicine, Fu Xing Hospital, Capital Medical University, Beijing, China; 4 Department of Critical Care Medicine, Jinyang Hospital Affiliated of Guizhou Medical University, Guiyang Second People’s Hospital, Guiyang, China; Phramongkutklao College of Medicine, THAILAND

## Abstract

**Background:**

This study assesses the incidence of ICU-acquired weakness (ICUAW) among patients using kidney replacement therapy (KRT) and explores the uncertain relationship between KRT and ICUAW in critically ill adult patients.

**Methods:**

A comprehensive search was conducted across PubMed, Embase, Web of Science, and the Cochrane Central Register of Controlled Trials up to June 10, 2024. Inclusion criteria encompassed randomized controlled trials (RCTs), as well as prospective and retrospective cohort studies that examined the correlation between KRT and ICUAW in adult ICU patients. Heterogeneity was evaluated using the *χ*^²^ and *I*^²^ statistics. Publication bias was assessed qualitatively via funnel plots and quantitatively using Begg’s and Egger’s tests.

**Results:**

A total of twelve cohort studies involving 2275 adult patients were included, with no RCTs meeting the criteria. The overall incidence of ICUAW was 49.5% in patients using KRT, compared to 34.8% in non-KRT controls. These studies collectively demonstrated a statistically significant association between KRT and a higher incidence of ICUAW (OR, 2.12; 95% CI, 1.34–3.34). Subgroup and sensitivity analyses reinforced this association, particularly in studies involving patients with clinical weakness, large sample sizes, and low risk of bias. However, studies focused on patients with abnormal electrophysiology and small sample sizes did not show this correlation. Despite the substantial findings, statistical heterogeneity was present. No significant publication bias was detected.

**Conclusion:**

This study highlights a significant association between KRT and an increased incidence of developing ICUAW.

## Introduction

Intensive care unit-acquired weakness (ICUAW) is a prevalent and debilitating neuromuscular complication that affects a substantial number of critically ill patients. ICUAW is characterized by severe muscle weakness and is associated with several adverse clinical outcomes, including prolonged mechanical ventilation, extended ICU and hospital stays, increased healthcare costs, and higher rates of ICU- and hospital-related mortality [1–3]. Beyond these acute impacts, ICUAW contributes to long-term functional impairments, complicating the rehabilitation process for survivors of critical illness. Acute kidney injury (AKI) is another frequent complication in critically ill patients, affecting nearly half of ICU admissions, with approximately 15% of these patients requiring kidney replacement therapy (KRT) to manage severe kidney dysfunction [4]. KRT, encompassing hemodialysis, continuous kidney replacement therapies, and other modalities, is a cornerstone treatment for supporting kidney function in critically ill patients. Due to the high incidence of AKI in ICUs, KRT has become a vital tool for managing severe kidney impairment. The relationship between KRT and ICUAW has garnered increasing interest in recent years. An early study [5] suggested that KRT might mitigate ICUAW by reducing the toxic burden of kidney failure, maintaining fluid and electrolyte balance, and optimizing hemodynamic stability. These findings proposed a potential protective effect of KRT, hypothesizing that improved kidney function and metabolic control might reduce factors contributing to muscle weakness. However, recent evidence challenges this view, indicating that ICU patients receiving KRT may be at a higher risk of developing ICUAW. This emerging perspective posits that KRT, while life-saving, could contribute to ICUAW through various mechanisms. Prolonged KRT exposure, particularly continuous therapies, may lead to electrolyte imbalances, fluid shifts, and other metabolic disturbances that exacerbate muscle dysfunction. Additionally, the severity of the illness necessitating KRT may independently elevate the risk of ICUAW, complicating the potential protective role of KRT. Given these complexities, there remains uncertainty regarding the relationship between KRT and ICUAW. Conflicting results from previous studies underscore the need for a more comprehensive evaluation. It is unclear whether KRT acts as a protective factor against ICUAW or if it inadvertently contributes to its development. This systematic review and meta-analysis aim to synthesize data from randomized controlled trials (RCTs) and prospective and retrospective cohort studies to assess the incidence of ICUAW among patients using KRT and explores the uncertain relationship between KRT and ICUAW in critically ill adult patients. By analyzing a broad range of studies, we hope to clarify this relationship and provide insights into how KRT may influence ICUAW risk in critically ill adults. These findings will help inform clinical practice and guide future research efforts to optimize care for ICU patients requiring KRT.

## Materials and methods

This study followed the Preferred Reporting Items for Systematic Reviews and Meta-Analyses (PRISMA) guidelines [6].

### Data sources and search strategy

A comprehensive search of the literature was conducted to identify relevant English-language studies published from the inception of each database through June 10, 2024. Databases searched included PubMed, Embase, Web of Science, and the Cochrane Central Register of Controlled Trials. The detailed search terms used for PubMed are provided in the Supplementary Material, and equivalent terms were applied to the other databases to ensure broad coverage. Additionally, manual searches of the reference lists in included studies and relevant review articles were conducted to capture any studies potentially missed by the electronic search.

### Selection criteria

Studies included in this review were RCTs and prospective or retrospective cohort studies that specifically examined the association between KRT and ICUAW in adult patients (aged ≥18 years). ICUAW diagnosis had to be based on established diagnostic criteria, such as manual muscle testing using the Medical Research Council (MRC) scale or diagnostic tools including electrophysiological studies and histopathological analyses of muscle or nerve tissue [7,8]. Studies focused on pediatric populations, those with insufficiently reported data, or patients with primary polyneuropathies or myopathies were excluded to ensure consistency in the patient populations studied.

### Study selection and data abstraction

Two independent reviewers (T.Y. and K.K.Z.) assessed the eligibility of studies through a structured selection process. Titles and abstracts were screened first, followed by full-text reviews of potentially relevant studies. Data extraction was performed using a standardized form that included details such as author names, publication year, study design, location, ICUAW incidence rates, participant numbers, neuromuscular evaluation methods, KRT treatment status, and mortality related to ICUAW. Any disagreements between reviewers were resolved through consensus or by involving a third reviewer (S.S.Y.) if needed. When data were incomplete or unclear, the original study authors were contacted for clarification.

### Study quality assessment

The methodological quality of each study was evaluated independently by two reviewers (T.Y. and K.K.Z.) using the Newcastle–Ottawa Scale.

### Data analysis

Meta-analysis was performed using Stata version 22.0 (StataCorp, College Station, TX, USA). Results were reported as odds ratios (ORs) with 95% confidence intervals (CIs), using the DerSimonian and Laird random effects model. Heterogeneity was assessed using the *χ*^²^ test, with a *P*-value ≤ 0.1 considered statistically significant, and the *I*^²^ statistic, with values above 50% indicating considerable heterogeneity. Subgroup and sensitivity analyses were conducted on studies based on clinical muscle testing, electrophysiology, study design (prospective vs. retrospective), sample size (excluding studies with fewer than 100 participants), and risk of bias (excluding studies with a Newcastle–Ottawa Scale score < 7). Publication bias was evaluated using Egger’s linear regression test and Begg’s rank correlation test for quantitative analysis, and funnel plots for qualitative assessment.

## Results

### Study search and selection

**Fig 1:**
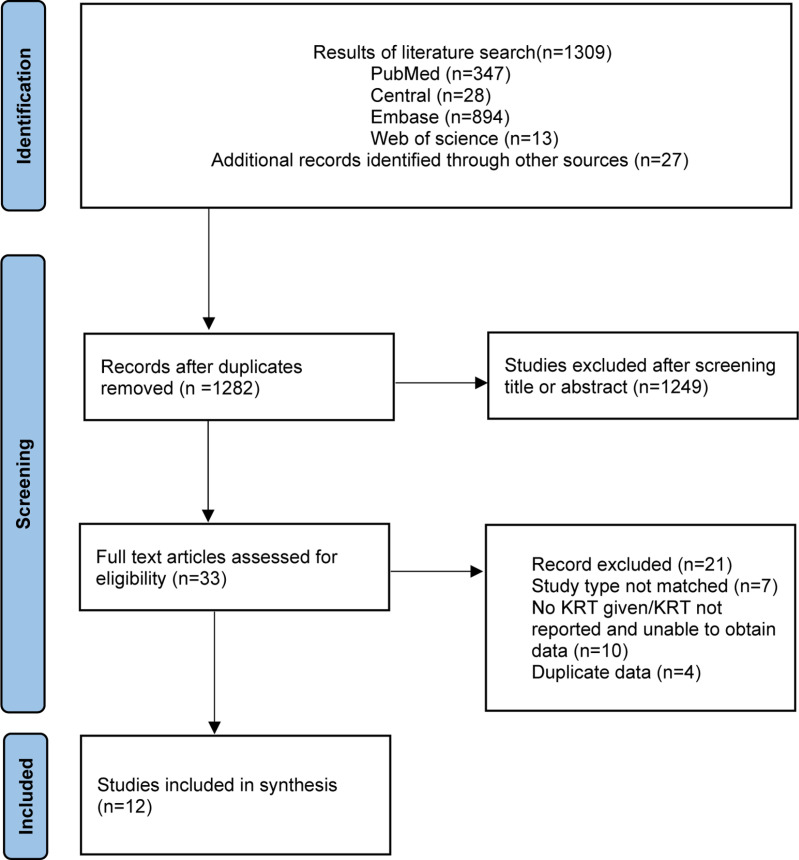
Flow diagram of study selection process.

The initial search of the databases yielded 1,309 citations, as illustrated in Fig 1. After removing duplicates and excluding irrelevant studies based on titles and abstracts, 33 articles underwent full-text review. Ultimately, 12 studies [5,9–19] met the inclusion criteria and were included in the final analysis.

### Study characteristics and quality

The characteristics of the included studies are summarized in Table 1. A total of 2,275 patients were covered, with nine studies [5,9,10,12,13,15–18] being prospective and three [11,14,19] retrospective cohort studies. No RCTs were included. ICUAW diagnosis methods varied, with eight studies [9–12,15,17–19] using the MRC scale and four [5,13,14,16] relying on electrophysiological methods. ICU mortality rates varied across the studies. Methodological quality assessment (Table 2) identified some bias, particularly in studies that did not use multivariable regression analysis to compare KRT effects [9,10,13,15,16,18,19] Moreover, several studies [15,19] did not specify whether outcome assessments were blinded. Inconsistent follow-up completeness was also noted in some studies [11,13,14,16].

**Table 1:** Characteristics of selected studies.

Study	Study Design	Country	Setting	Population	n	Examination	ICUAW	Use of KRT ^a^	ICU Mortality ^a^
Lu, C. 2024[18]	Prospective	China	ICU	ICU-LOS ≥ 7 d and MV > 48 h	264	Clinical	116	19 vs 23	NR
Liu, J. 2024 [19]	Retrospective	China	ICU	Sepsis	264	Clinical	114	53 vs 57	NR
Watanabe, S. 2023 [11]	Retrospective	Japan	ICU	MV > 48 h	143	Clinical	62	25 vs 15	NR
Yang, Z. 2023 [9]	Prospective	China	ICU	ICU-LOS > 24h	280	Clinical	40	9 vs 50	NR
Yamada, K. 2023 [10]	Prospective	Japan	ICU	MV ≥ 48 h and SARS-CoV-2	157	Clinical	80	8 vs 1	NR
Schmidt, D. 2022 [12]	Prospective	Brazil	ICU	ICU-LOS ≥ 72 h and SARS-CoV-2	75	Clinical	28	13 vs 6	0% VS 2.1%
Frithiof, R. 2021 [13]	Prospective	Sweden	ICU	SARS-CoV-2	111	EMG	11	7 vs 8	20% vs 27%
Hermans, G. 2009 [14]	Retrospective	Belgium	ICU	MV > 7 d	541	EMG	301	118 vs 72	NR
Nanas, S. 2008 [15]	Prospective	Greece	ICU	LOS > 10 d	185	Clinical	44	16 vs 31	36% vs 20%
De Jonghe, B. 2002 [17]	Prospective	France	ICU	MV > 7 d and awake	95	Clinical	24	8 vs 9	17% vs 6%
Garnacho-Montero, J. 2001 [5]	Prospective	Spain	ICU	MV > 10 d and sepsis with MOF	73	EMG	50	10 vs 11	66% vs 52.2%
Campellone, J. V. 1998 [16]	Prospective	America	ICU	Hospital LOS ≥ 14 d or MV ≥ 7 d	87	EMG	7	4 vs 13	NR

ICUAW, intensive care unit-acquired weakness; KRT, kidney replacement therapy; ICU, intensive care unit; MV, mechanical ventilation; NR, not reported; LOS, length of stay; EMG, Electromyography; MOF, multiple organ failure.

^a^comparison between ICUAW and no ICUAW.

**Table 2:** Methodology and reporting assessment.

Newcastle-Ottawa quality assessment scale for cohort studies									
Studies	Selection				Comparability	Outcome			Score
	Exposed representative?	Non-exposed representative?	Ascertainment of exposure	Outcome of interest not present at start		Assessment of outcome	Adequate duration of follow-up	Completeness of follow-up	
Lu, C. 2024[18]	Y	Y	Y	Y	N, N	Y	Y	Y	7
Liu, J. 2024 [19]	Y	Y	Y	Y	Y, N	N	Y	Y	7
Watanabe, S. 2023 [11]	Y	Y	Y	Y	Y, Y	Y	N	N	7
Yang, Z. 2023 [9]	Y	Y	Y	Y	N, N	Y	Y	Y	7
Yamada, K. 2023 [10]	Y	Y	Y	Y	N, N	Y	Y	Y	7
Schmidt, D. 2022 [12]	Y	Y	Y	Y	Y, Y	Y	Y	Y	9
Frithiof, R. 2021 [13]	Y	Y	Y	Y	Y, N	Y	N	N	6
Hermans, G. 2009 [14]	Y	Y	Y	Y	Y, Y	Y	N	Y	8
Nanas, S. 2008 [15]	Y	Y	Y	Y	N, N	N	Y	Y	6
De Jonghe, B. 2002 [17]	Y	Y	Y	Y	Y, Y	Y	Y	Y	9
Garnacho-Montero, J. 2001 [5]	Y	Y	Y	Y	Y, Y	Y	Y	Y	9
Campellone, J. V. 1998 [16]	Y	Y	Y	Y	N, N	Y	Y	N	6

Y - criteria satisfied, N - criteria not satisfied

### KRT and ICUAW

**Fig 2:**
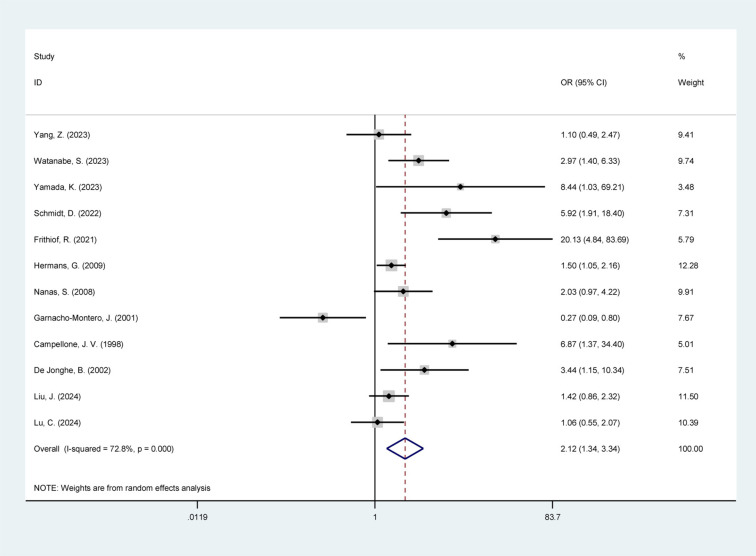
Meta-analysis of included studies.

The overall incidence of ICUAW among the included studies was 38.5%; patients receiving KRT had a higher incidence of ICUAW (49.5%) compared to controls (34.8%). As illustrated in Fig 2, the meta-analysis of the 12 included studies [5,9–19] revealed a significant association between KRT and an increased likelihood of ICUAW (OR 2.12; 95% CI 1.34–3.34; *P* < 0.01). Due to significant heterogeneity (*I*² = 72.8%, *P* < 0.01), a random-effects model was applied.

### Subgroup analyses

**1. Clinical assessment versus electrophysiology**

Eight studies [9–12,15,17–19] that used clinical muscle testing showed a significant association between KRT and increased clinical weakness (OR 2.02; 95% CI 1.33–3.07; *P* < 0.01), with a random effects model considering the heterogeneity (*I*² = 50.6%, *P* = 0.05). The incidence of ICUAW was substantially higher in patients using KRT (44.0%) compared to those in the control group (31.9%). In contrast, the four studies [5,13,14,16] that used electrophysiological assessments did not show a significant relationship (OR 2.48; 95% CI 0.56–10.98; *P* = 0.23) with an incidence rate of 57.2% in the KRT group versus 40.4% in the control group. Data were pooled using a random effects model considering the observed heterogeneity (*I*² = 88.4%, P < 0.01) (Table 3).

**Table 3:** Subgroup and sensitivity analyses.

Analyses	Study	n	*I*^2^(%)	Ph	OR	95%CI	Pe	Incidence KRT	Incidence control
Primary analysis	5, 9-19	2275	72.8%	<0.01	2.12	1.34-3.34	<0.01	49.5%	34.8%
AKI	9, 12, 17	450	61.6%	0.07	2.18	0.86-5.54	0.10	34.4%	16.9%
Diagnostic method									
Clinical assessment	9-12, 15, 17-19	1463	50.6%	0.05	2.02	1.33-3.07	<0.01	44.0%	31.9%
Electrophysiology	5, 13, 14, 16	812	88.4%	<0.01	2.48	0.56-10.98	0.23	57.2%	40.4%
Study size									
Large sample	9-11, 13-15, 18, 19	1945	65.1%	<0.01	1.95	1.27-3.01	<0.01	49.8%	35.8%
Small sample	5, 12, 16, 17	330	85%	<0.01	2.39	0.51-11.29	0.27	47.3%	28.9%
Study type									
Prospective studies	5, 9, 10, 12, 13, 15-18	1327	78.5%	<0.01	2.52	1.19-5.35	0.02	38.2%	28.3%
Retrospective studies	11, 14, 19	948	31.5%	0.23	1.66	1.17-2.36	<0.01	57.6%	46.2%
Sensitivity analysis	5, 9-12, 14, 17-19	1749	67.9%	<0.01	1.65	1.05-2.59	0.03	51.0%	40.2%

*I*^2^, *I*-squared statistic test for heterogeneity; Ph, P value for test of heterogeneity; OR, odds ratio; CI, confidence intervals; Pe, P value for the effect estimate for each subgroup; KRT, kidney replacement therapy.

**2. Prospective vs retrospective cohort studies**

Nine prospective studies [5,9,10,12,13,15–18] demonstrated a higher unadjusted incidence rate of 38.2% in the KRT group compared to 28.3% in the control group. A random effects model was employed for this analysis due to significant heterogeneity (*I*² = 78.5%, *P* < 0.01), revealing a significant association between KRT and increased ICUAW incidence (OR 2.52; 95% CI 1.19–5.35; *P* = 0.02). Similarly, three retrospective studies [11,14,19] also found a significant association (OR 1.66; 95% CI 1.17–2.36; *P* < 0.01), with an incidence rate of 57.6% in the KRT group versus 46.2% in the control group. Data were pooled using a random effects model, accounting for moderate heterogeneity (*I*² = 31.5%, *P* = 0.23) (Table 3).

**3. Sample sizes (n ≥ 100 versus n < 100)**

The eight studies [9–11,13–15,18,19] with sample sizes over 100 revealed a significant association between KRT and ICUAW (OR 1.95; 95% CI 1.27–3.01; *P* < 0.01). Data were pooled using a random effects model considering the observed heterogeneity (*I*² = 65.1%, *P* < 0.01). ICUAW incidence was higher in the KRT group (49.8%) compared to controls (38.5%). However, the four studies [5,12,16,17] with smaller sample sizes (n < 100) did not demonstrate a significant association (OR 2.39; 95% CI 0.51–11.29; *P* = 0.27). A random effects model was employed for this analysis due to significant heterogeneity (*I*² = 85%, *P* < 0.01) (Table 3).

**4. AKI and ICUAW**

Of the included studies, three studies [9,12,17] reported the incidence of ICUAW in patients with AKI. However, patients with AKI did not demonstrate a significantly higher incidence of ICUAW at 34.4%, compared to 16.9% in non-AKI controls. Analysis of three studies did not show a statistically significant association between AKI and an increased likelihood of developing ICUAW (OR 2.18; 95% CI, 0.86–5.54; *P* = 0.10), with a random effects model considering the heterogeneity (*I*² = 61.6%, *P* = 0.07) (Table 3).

### Sensitivity analysis

Excluding studies [13,15,16] with a high risk of bias, the analysis still demonstrated a significant association between KRT and ICUAW (OR 1.65; 95% CI 1.05–2.59; *P* = 0.03) (Table 3). The random-effects model was employed, considering the observed heterogeneity (*P* < 0.01; *I*² = 67.9%). The incidence of ICUAW escalated to 51.0% in the KRT group, against 40.2% in the control group (Table 3).

### Illness severity

Five studies [10,11,15,18,19] examined whether Acute Physiology and Chronic Health Evaluation (APACHE) II score was an independent risk factor for ICUAW, and the overall effect size (OR, 1.12; 95%CI, 1.06–1.18; *P* < 0.01). The random-effects model was employed, considering the observed heterogeneity (P = 0.02; *I*² = 66.9%).

### Heterogeneity

Significant heterogeneity was observed across the included studies, which can be attributed to variations in diagnostic methods, patient populations, and study designs. To address this variability, a random-effects model was employed for all analyses. The studies utilized two distinct diagnostic approaches and two primary study design types. Additionally, sample sizes varied considerably, with studies categorized as small or large based on a cutoff of 100 participants. Due to this heterogeneity, the review conducted three comparative analyses: clinical assessment versus electrophysiology, prospective versus retrospective cohort studies, and sample size analysis (n ≥ 100 versus n < 100).

### Assessment of publication biases

**Fig 3:**
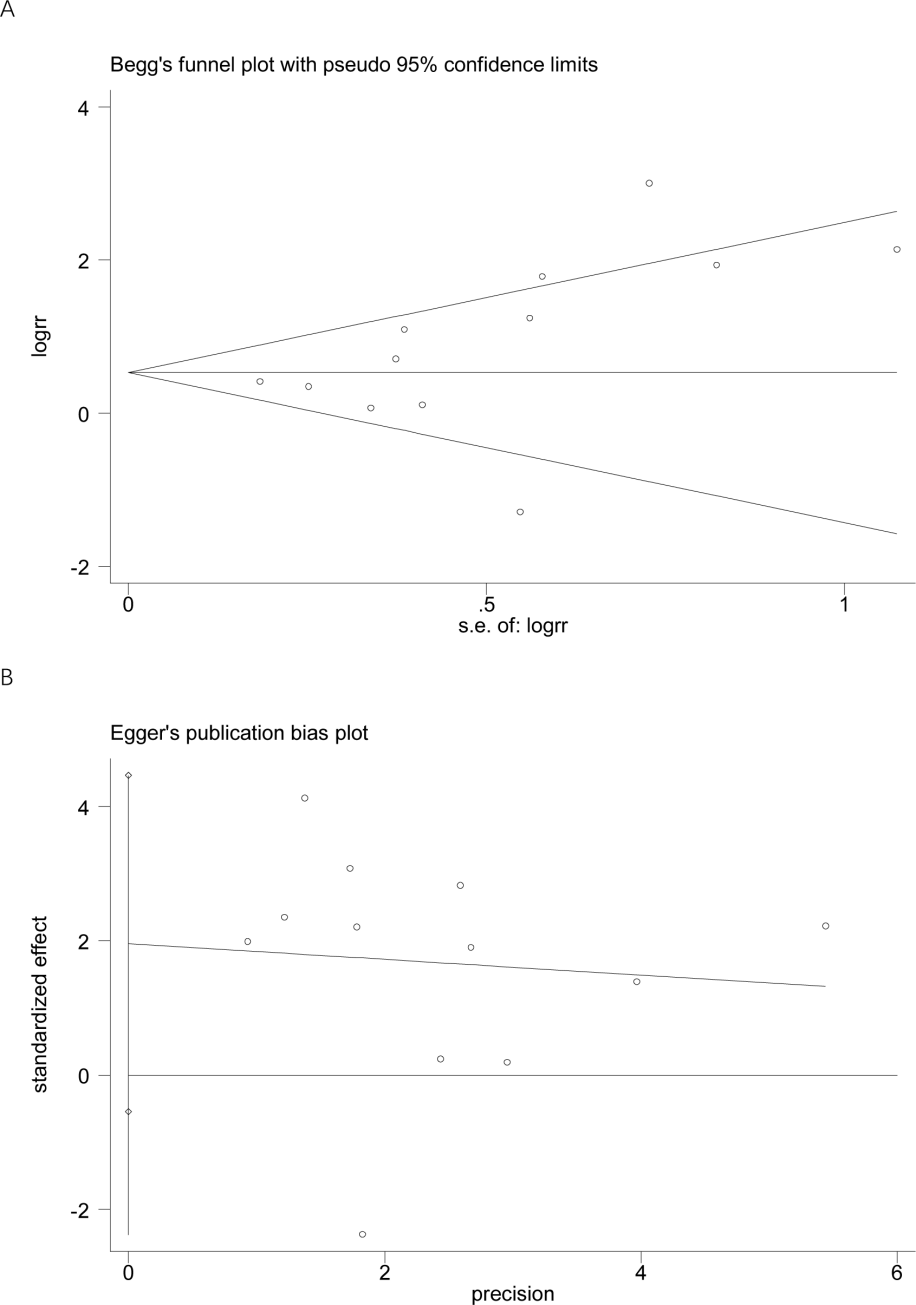
Funnel plots of the included studies.

No significant publication bias was detected based on visual inspection of funnel plots and the results of Egger’s test (*t* = 1.74, *P* = 0.11) and Begg’s test (*z* = 1.85, *P* = 0.06) (Fig 3).

## Discussion

This comprehensive systematic review and meta-analysis provide valuable insights into the complex relationship between KRT and the development of ICUAW, and the incidence of ICUAW in critically ill patients treated with kidney replacement therapy. Our findings suggest that KRT is a significant risk factor for increasing odds of ICUAW, with patients undergoing KRT potentially experiencing skeletal muscle dysfunction due to a variety of physiological and metabolic mechanisms. One plausible explanation for this increased risk is the non-selective removal of water-soluble molecules during hemodialysis and hemofiltration, including essential amino acids, peptides, and small proteins. These molecules are vital for maintaining muscle integrity and function, and their depletion during KRT may contribute to muscle wasting and weakness [20]. While some studies have suggested amino acid supplementation as a possible intervention, evidence regarding its efficacy remains inconclusive. This uncertainty raises important questions about the optimal nutritional management of patients undergoing KRT, especially concerning ICUAW prevention. Although current clinical guidelines recommend increased protein supplementation for patients on KRT [21], the lack of robust evidence supporting this approach highlights the need for further research into personalized nutritional strategies that better address the metabolic needs of these patients. Another critical factor contributing to ICUAW is prolonged immobilization or bedrest, often necessitated by the severity of illness and the complexities of KRT. Immobility accelerates muscle atrophy through mechanical silencing and disuse, compounding the muscle dysfunction already present in critically ill patients. In this context, early mobilization, physical rehabilitation, and structured exercise programs [22] have proven to be essential interventions in mitigating the adverse effects of immobility. Several studies have demonstrated the feasibility and safety of early mobilization in ICU patients, including those with vascular catheters and undergoing KRT [23–25]. Early rehabilitation not only preserves muscle strength but also improves overall outcomes, such as reduced ICU stays and better functional recovery. Despite the logistical challenges posed by KRT, such as continuous monitoring and equipment management, recent research supports integrating early mobilization protocols in patients on KRT. This underscores the importance of multidisciplinary collaboration in the ICU, ensuring that physical therapy and mobilization efforts are synchronized with kidney support therapies.

AKI is a critical clinical syndrome characterized by the rapid deterioration of renal function, leading to significant structural and functional impairments of the kidneys. It is commonly observed in acute illness settings and is strongly linked to adverse clinical outcomes [26–28]. Analysis of the included studies revealed no statistically significant association between AKI and an increased risk of developing ICUAW.

Regarding illness severity, the APACHE II score, a validated tool for assessing acute physiological abnormalities and predicting ICU mortality, was utilized to evaluate disease severity. The pooled results demonstrated that patients with higher APACHE II scores had a greater likelihood of developing ICUAW. Furthermore, the APACHE II score, as a measure of illness severity, was identified as an independent risk factor for ICUAW.

Subgroup analyses in our review underscore the nuanced relationship between KRT and ICUAW. The significant association between KRT and muscle weakness was more pronounced in patients diagnosed using clinical muscle testing (e.g., MRC scale) compared to those diagnosed through electrophysiological methods. However, within the electrophysiology subgroup, the incidences of ICUAW in both the KRT and control groups were higher than those observed in the clinical assessment subgroup. ICUAW is fundamentally a condition characterized by clinically detectable weakness, and clinical examinations are generally simpler and more practical to perform than electrophysiological assessments. Nevertheless, clinical examinations are often limited in the early stages of the disease due to impaired levels of consciousness or attentiveness in critically ill patients. This suggests that electrophysiological tests may be more sensitive in detecting ICUAW at advanced stages, whereas clinical examinations may fail to capture subclinical muscle dysfunction in its early phases. These findings highlight concerns regarding the limitations of early detection through clinical examinations, as they may not identify neuromuscular impairment until weakness becomes overt. These considerations may provide an alternative explanation for the observed differences in outcomes.

This review demonstrates methodological robustness through consistent findings across subgroup analyses, with results remaining stable regardless of study design, sample size, or risk of bias level, consistently supporting the association between KRT and ICUAW. However, several limitations merit careful consideration. The predominant reliance on observational studies, particularly cohort designs, coupled with the absence of RCTs, constrains our ability to establish causal relationships. Methodological concerns primarily stem from the widespread use of univariate analyses in most included studies, potentially leading to overestimation of effects due to inadequate control of confounding variables. Among the limited number of studies reporting multivariate analysis results for KRT, the findings were inconsistent, with one study [5] identifying KRT as protective, another [12] suggesting potential harm, and a third [11] finding no significant association. Additional methodological limitations include potential detection bias arising from unblinded outcome assessment in several studies and possible attrition bias due to inconsistent follow-up protocols. The review also identified significant knowledge gaps, particularly regarding the role of AKI in ICUAW development, with insufficient data available to explore this relationship in non-KRT patients. Despite these limitations, the robustness of the primary findings is supported by sensitivity analyses that maintained significant associations after excluding high-risk studies, while publication bias assessments using funnel plots and statistical tests (Egger’s and Begg’s) showed no significant bias. The persistent heterogeneity observed across studies, even after subgroup analyses by study design, diagnostic methods, and sample size, underscores the complexity of this clinical relationship. Moving forward, research should prioritize the implementation of RCTs and prospective multivariate designs to better account for confounding variables. Standardization of outcome assessment protocols, implementation of comprehensive follow-up procedures, and detailed investigation of AKI’s contribution to ICUAW pathophysiology, particularly in milder forms and non-KRT management scenarios, will be essential for advancing our understanding of this complex clinical relationship and informing evidence-based management strategies for critically ill patients.

## Conclusion

This systematic review and meta-analysis establish a significant association between KRT and the increased incidence of developing ICUAW in critically ill patients. Future research should focus on RCTs or prospective cohort studies by performing multivariable adjustment for confounders to identify the associations between the KRT and ICUAW. By deepening our understanding of the complex interactions between critical illness, KRT, and muscle weakness, we can enhance the quality of care and long-term recovery for ICU survivors. Besides, multidisciplinary care strategies, including tailored nutritional support, early mobilization and rehabilitation protocols, may be beneficial for mitigating the harmful effects of ICUAW and improving patient outcomes.

## Supporting information

S1 Table Excluded studies and reasons for exclusion. (DOCX)

S2 Table PRISMAChecklist. (DOC)

S3 Table Data extraction of included studies. (DOCX)

S1 File PubMed search strategy. (DOCX)
