# Supplementary material for: Incidence of intensive care unit acquired weakness in critically ill patients treated with kidney replacement therapy: A systematic review and meta-analysis
Source: PLoS One. 2025 May 15;20(5):e0323874. doi: 10.1371/journal.pone.0323874 (PMC12080829; doi:10.1371/journal.pone.0323874)
Supplement: S1 Table — (DOCX) [file pone.0323874.s001.docx]

**S1 Table. Excluded studies and reasons for exclusion**

| **Studies** | **Justification** |
| --- | --- |
| Abbenhaus & Kamineni, 2020; | No reported data on KRT |
| Abdelmalik & Rakocevic, 2017; | No reported data on KRT |
| Abdelmonem, Backx, Vale, Healy, & Williams, 2022; | No reported data on KRT |
| Abdi et al., 2022; | No reported data on KRT |
| Abouyannis et al., 2021; | No reported data on KRT |
| Actrn, 2016; | No reported data on KRT |
| Adamski, Koivuranta, & Leppänen, 2006; | No reported data on KRT |
| Adu, Cheshire, Riga, Hamady, & Bicknell, 2012; | No reported data on KRT |
| Aebi et al., 2010; | No reported data on KRT |
| Agarwal & Divatia, 2019; | No reported data on KRT |
| Agbaht, Bitik, Piskinpasa, Bayraktar, & Topeli, 2009; | No reported data on KRT |
| Aggarwal, Pillai, Billington, Schema, & Berry, 2022; | No reported data for KRT and ICUAW |
| Agostini & Bianchin, 2003; | No reported data for KRT and ICUAW |
| Agrawal & Kanagasundaram, 2015; | No reported data on KRT |
| B. Ahmed, Hanna, & Nichol, 2015; | No reported data on KRT |
| Z. Ahmed, Barefah, Wasi, Jones, & Ramsay, 2019; | No reported data on KRT |
| Akers et al., 2015; | No reported data for KRT and ICUAW |
| Akhtar & Deshmukh, 2021; | No reported data for KRT and ICUAW |
| Akinremi, Erinle, & Hamzat, 2019; | No reported data on KRT |
| Akkanti et al., 2022; | No reported data on KRT |
| Al-Lahham & Lacomis, 2022; | No reported data on KRT |
| Al Ali, Singh, Filler, & Ramsi, 2021; | No reported data on KRT |
| Al Khaldi, Gulreez, Abdelhamid, & Louri, 2023; | No reported data on KRT |
| Alawad, Zara, Elgohari, Ibrahim, & Abdel Hadi, 2023; | No reported data on KRT |
| Albrecht et al., 2024; | No reported data on KRT |
| Albuquerque et al., 2018; | No reported data on KRT |
| Alfonzo, Fox, Imrie, Roditi, & Young, 2006; | No reported data on KRT |
| Alfred et al., 2005; | No reported data on KRT |
| Alhamad et al., 2012; | No reported data on KRT |
| Ali et al., 2022; | Cross-sectional study |
| Alobaidi, Backdash, & El-Menyar, 2016; | No reported data for KRT and ICUAW |
| AlOtaibi et al., 2021; | No reported data for KRT and ICUAW |
| Alowayshiq et al., 2018; | No reported data for KRT and ICUAW |
| Altintepe et al., 2007; | No reported data for KRT and ICUAW |
| Alwardt et al., 2013; | No reported data for KRT and ICUAW |
| Amaducci et al., 2023; | No reported data for KRT and ICUAW |
| Amery, Davies, & Forni, 2015; | No reported data for KRT and ICUAW |
| Amiri, 2015; | No reported data for KRT and ICUAW |
| Ammayappan et al., 2022; | No reported data for KRT and ICUAW |
| Anand et al., 2023; | No reported data for KRT and ICUAW |
| Andrade, de Francesco Daher, & Seguro, 2008; | No reported data for KRT and ICUAW |
| Angraje, Sekar, Mishra, & Matcha, 2021; | No reported data for KRT and ICUAW |
| Angriman, Muttalib, Lamontagne, & Adhikari, 2023; | No reported data for KRT and ICUAW |
| Angurana, Jayashree, Bansal, Singhi, & Nallasamy, 2018; | No reported data for KRT and ICUAW |
| Annangi & Pasha, 2021; | No reported data for KRT and ICUAW |
| Anonymous, 2019; | No reported data for KRT and ICUAW |
| Antonio, Knorst, & Teixeira, 2018; | No reported data for KRT and ICUAW |
| Anvar, Bhaskar, Chand, Kalaburgi, & Shaik, 2022; | No reported data for KRT and ICUAW |
| Appelman, van-Der-Heijden, Meenks, Janssen, & le Noble, 2023; | No reported data for KRT and ICUAW |
| Arabi et al., 2015; | No reported data for KRT and ICUAW |
| Arias-Rivera, Raurell-Torredà, Thuissard-Vasallo, Andreu-Vázquez, & Hodgson, 2020; | No reported data for KRT and ICUAW |
| Arjmand, Shahriarirad, Shenavandeh, & Fallahi, 2022; | No reported data for KRT and ICUAW |
| Armstrong, McCurdy, & Heavner, 2019; | No reported data for KRT and ICUAW |
| Arumugam, Balakrishnan, & Parchani, 2011; | No reported data for KRT and ICUAW |
| Asarani, Paddison, Walker, Downie, & Wheeler, 2021; | No reported data for KRT and ICUAW |
| Asfar et al., 2017; | No reported data for KRT and ICUAW |
| Aslan et al., 2023; | No reported data for KRT and ICUAW |
| Atan et al., 2015; | No reported data for KRT and ICUAW |
| Atemnkeng, Aguilar, Gupta, Chugh, & Klein, 2023; | No reported data for KRT and ICUAW |
| Atmis et al., 2023; | No reported data for KRT and ICUAW |
| Attwell et al., 2022; | No reported data for KRT and ICUAW |
| Augustin et al., 2006; | No reported data for KRT and ICUAW |
| Avasarala, Qureshi, Waldron, Mukherjee, & Reddy, 2019; | No reported data for KRT and ICUAW |
| Aylward et al., 2019; | No reported data for KRT and ICUAW |
| Ayoǧlu et al., 2009; | No reported data for KRT and ICUAW |
| Azevedo et al., 2023; | No reported data for KRT and ICUAW |
| Baby, George, & Osahan, 2021; | No reported data for KRT and ICUAW |
| Bache, Taggart, & Gilhooly, 2011; | No reported data for KRT and ICUAW |
| Bachoumas et al., 2015; | No reported data for KRT and ICUAW |
| Bagnato et al., 2020; | No reported data for KRT and ICUAW |
| Bagshaw, Neyra, Tolwani, & Wald, 2023; | No reported data for KRT and ICUAW |
| Bailuni Neto et al., 2022; | No reported data for KRT and ICUAW |
| Balan et al., 2021; | No reported data for KRT and ICUAW |
| Baldwin & Sran, 2010; | No reported data for KRT and ICUAW |
| Ballvé et al., 2021; | No reported data for KRT and ICUAW |
| Bamber, Rudge, & Vercueil, 2019; | No reported data for KRT and ICUAW |
| Barad, 2018; | No reported data for KRT and ICUAW |
| Barea-Mendoza et al., 2022; | No reported data for KRT and ICUAW |
| Barth et al., 2023; | No reported data for KRT and ICUAW |
| Bartoszko et al., 2022; | No reported data for KRT and ICUAW |
| Basnet, Pantha, Acharya, Shrestha, & Kafle, 2019; | No reported data for KRT and ICUAW |
| Batt, Herridge, & Dos Santos, 2017; | No reported data for KRT and ICUAW |
| Batt, Mathur, & Katzberg, 2017; | No reported data for KRT and ICUAW |
| Bax et al., 2021; | No reported data for KRT and ICUAW |
| Belay & Nusair, 2013; | No reported data for KRT and ICUAW |
| Belin, Clairet, Chocron, Capellier, & Piton, 2017; | No reported data for KRT and ICUAW |
| Bell, Chawla, & Wald, 2017; | No reported data for KRT and ICUAW |
| Bellaver et al., 2023; | No reported data for KRT and ICUAW |
| Belliere et al., 2017; | No reported data for KRT and ICUAW |
| Bellomo, 2011; | No reported data for KRT and ICUAW |
| Bender, Chew, Lin, & Athan, 2018; | No reported data for KRT and ICUAW |
| Berenguer et al., 2020; | No reported data for KRT and ICUAW |
| Bergmann et al., 2020; | No reported data for KRT and ICUAW |
| Berlit et al., 2020; | No reported data for KRT and ICUAW |
| Bertoni et al., 2022; | No reported data for KRT and ICUAW |
| Bertucci et al., 2019; | No reported data for KRT and ICUAW |
| Betters et al., 2017; | No reported data for KRT and ICUAW |
| Bhadra et al., 2019; | No reported data for KRT and ICUAW |
| Biberoğlu, Cakmak, Ozkan, Ikizceli, & Ipekci, 2021; | No reported data for KRT and ICUAW |
| Bickenbach et al., 2024; | No reported data for KRT and ICUAW |
| Bilbault et al., 2007; | No reported data for KRT and ICUAW |
| Binder et al., 2020; | No reported data for KRT and ICUAW |
| Boedecker et al., 2021; | No reported data for KRT and ICUAW |
| Boelens, Strookappe, Vasse, Mensink, & van Zanten, 2022; | No reported data for KRT and ICUAW |
| Boërio et al., 2018; | No reported data for KRT and ICUAW |
| Bonanni et al., 2020; | No reported data for KRT and ICUAW |
| Borré-Naranjo, Rodríguez-Yánez, Almanza-Hurtado, Martínez-Ávila, & Dueñas-Castell, 2022; | No reported data for KRT and ICUAW |
| Boue et al., 2014; | No reported data for KRT and ICUAW |
| Bouju et al., 2017; | No reported data for KRT and ICUAW |
| Bragança et al., 2019; | No reported data for KRT and ICUAW |
| Branea et al., 2023; | No reported data for KRT and ICUAW |
| Braune et al., 2013; | No reported data for KRT and ICUAW |
| Brener & Brenner, 2021; | No reported data for KRT and ICUAW |
| Bridi, Balbi, Neves, & Ponce, 2014; | No reported data for KRT and ICUAW |
| Bright et al., 2023; | No reported data for KRT and ICUAW |
| Brivet, Slama, Prat, & Jacobs, 2011; | No reported data for KRT and ICUAW |
| Brock et al., 2018; | No reported data for KRT and ICUAW |
| Brown et al., 2023; | No reported data for KRT and ICUAW |
| Brummel et al., 2020; | No reported data for KRT and ICUAW |
| Buccione et al., 2021; | No reported data for KRT and ICUAW |
| Buitendag et al., 2021; | No reported data for KRT and ICUAW |
| Bulfon et al., 2019; | No reported data for KRT and ICUAW |
| Burgess, 2022; | No reported data for KRT and ICUAW |
| Burke et al., 2021; | No reported data for KRT and ICUAW |
| Burton, Lin, Said, & Gabriel, 2019; | No reported data for KRT and ICUAW |
| Busch et al., 2014; | No reported data for KRT and ICUAW |
| Byler, Harrison, & Fell, 2021; | No reported data for KRT and ICUAW |
| Byrne, Wheen, & O'Connor, 2021; | No reported data for KRT and ICUAW |
| Cabañes-Martínez et al., 2020; | No reported data for KRT and ICUAW |
| Cafardi, Haas, Lamarre, & Feinberg, 2021; | No reported data for KRT and ICUAW |
| Cambournac, Moumadah, Berny, & España, 2023; | No reported data for KRT and ICUAW |
| Campos et al., 2022; | No reported data for KRT and ICUAW |
| Candan, Elibol, & Abdullahi, 2020; | No reported data for KRT and ICUAW |
| Candela et al., 2020; | No reported data for KRT and ICUAW |
| Cantwell et al., 2017; | No reported data for KRT and ICUAW |
| H. Y. Cao et al., 2023; | No reported data for KRT and ICUAW |
| Cao et al., 2020; | No reported data for KRT and ICUAW |
| Y. Cao et al., 2023; | No reported data for KRT and ICUAW |
| Capdevila et al., 2023; | No reported data for KRT and ICUAW |
| Carberry et al., 2021; | No reported data for KRT and ICUAW |
| Carr et al., 2012; | No reported data for KRT and ICUAW |
| Cartotto et al., 2023; | No reported data for KRT and ICUAW |
| Cassim, Soni, & Murphy, 2021; | No reported data for KRT and ICUAW |
| Castejón-Hernández, Reynaga-Sosa, Navarro-Aguirre, & Vilamala-Bastarras, 2021; | No reported data for KRT and ICUAW |
| Castelli et al., 2023; | No reported data for KRT and ICUAW |
| Cebeci et al., 2022; | No reported data for KRT and ICUAW |
| Cecchini et al., 2022; | No reported data for KRT and ICUAW |
| A. Cetinkaya, Aydin, Sirakaya, & Yilmaz, 2020; | No reported data for KRT and ICUAW |
| R. Cetinkaya, Uyanik, Keles, & Bilen, 2009; | No reported data for KRT and ICUAW |
| Chabowski, Bieganski, Kobecki, Szponder, & Janczak, 2019; | No reported data for KRT and ICUAW |
| Chakraborty & Majhail, 2020; | No reported data for KRT and ICUAW |
| C. C. Chang et al., 2022; | No reported data for KRT and ICUAW |
| K. Y. Chang et al., 2014; | No reported data for KRT and ICUAW |
| Chani, Abouzahir, Haimeur, Drissi Kamili, & Mion, 2012; | No reported data for KRT and ICUAW |
| Chanques, Drouot, & Payen, 2018; | No reported data for KRT and ICUAW |
| Chapman, Shah, & D’Angelo, 2023; | No reported data for KRT and ICUAW |
| Chavez et al., 2019; | No reported data for KRT and ICUAW |
| Cheah et al., 2019; | No reported data for KRT and ICUAW |
| B. Chen et al., 2020; | No reported data for KRT and ICUAW |
| H. Chen, Aroch, & Segev, 2018; | No reported data for KRT and ICUAW |
| J. Chen et al., 2023; | No reported data for KRT and ICUAW |
| L. J. Chen et al., 2013; | No reported data for KRT and ICUAW |
| L. L. Chen, Hsu, Tian, & Fang, 2005; | No reported data for KRT and ICUAW |
| P. H. Chen et al., 2022; | No reported data for KRT and ICUAW |
| S. Chen et al., 2019; | No reported data for KRT and ICUAW |
| X. Chen, Lei, Xu, Zhou, & Huang, 2022; | No reported data for KRT and ICUAW |
| Y. Chen et al., 2023; | No reported data for KRT and ICUAW |
| Y. C. Chen, Fang, Chang, & Chang, 2000; | No reported data for KRT and ICUAW |
| Y. G. Chen et al., 2015; | No reported data for KRT and ICUAW |
| Z. Chen et al., 2021; | No reported data for KRT and ICUAW |
| Cherunghattil, Panda, Karim, & Dhawan, 2023; | No reported data for KRT and ICUAW |
| Chetram, Ahmad, Farid, & Sood, 2021; | No reported data for KRT and ICUAW |
| Chhetri et al., 2022; | No reported data for KRT and ICUAW |
| Chhetri et al., 2019; | No reported data for KRT and ICUAW |
| Chia, Barrett, Patel, & Soni, 2021; | No reported data for KRT and ICUAW |
| Chidambaram, Bhowmick, Parameswaran, & Gunasekaran, 2021; | No reported data for KRT and ICUAW |
| Chillura et al., 2020; | No reported data for KRT and ICUAW |
| Chirinos, Neyra, Patel, & Rodan, 2014; | No reported data for KRT and ICUAW |
| Chiu et al., 2019; | No reported data for KRT and ICUAW |
| Chiumello et al., 2012; | No reported data for KRT and ICUAW |
| Cho et al., 2022; | No reported data for KRT and ICUAW |
| Chohan, Ash, & Senior, 2018; | No reported data for KRT and ICUAW |
| Choi et al., 2015; | No reported data for KRT and ICUAW |
| Choleva, 2010; | No reported data for KRT and ICUAW |
| Choreño-Parra et al., 2021; | No reported data for KRT and ICUAW |
| F. C. Chow, Edlow, Frosch, Copen, & Greer, 2011; | No reported data for KRT and ICUAW |
| Y. W. Chow, Lim, & Hooi, 2007; | No reported data for KRT and ICUAW |
| Chowdhry, Agrawal, & S, 2021; | No reported data for KRT and ICUAW |
| Chu et al., 2003; | No reported data for KRT and ICUAW |
| Chua, Zhou, Lingegowda, Kwa, & Lee, 2014; | No reported data for KRT and ICUAW |
| Cippà, Auinger, Wüthrich, & Segerer, 2015; | No reported data for KRT and ICUAW |
| Clayton, Freeman-Sanderson, & Walker, 2024; | No reported data for KRT and ICUAW |
| Cline, Jajosky, Shikle, & Bollag, 2018; | No reported data for KRT and ICUAW |
| Coban, Yildizdas, Horoz, Aslan, & Bayazit, 2022; | No reported data for KRT and ICUAW |
| Coenen, Tran, De Haan, & De Man, 2017; | No reported data for KRT and ICUAW |
| Cointault et al., 2004; | No reported data for KRT and ICUAW |
| Cole et al., 2022; | No reported data for KRT and ICUAW |
| Collange et al., 2020; | No reported data for KRT and ICUAW |
| Colombo et al., 2021; | No reported data for KRT and ICUAW |
| Colonna et al., 2020; | No reported data for KRT and ICUAW |
| B. Connolly et al., 2018; | No reported data for KRT and ICUAW |
| M. Connolly et al., 2018; | No reported data for KRT and ICUAW |
| Cools et al., 2022; | No reported data for KRT and ICUAW |
| Coopersmith et al., 2012; | No reported data for KRT and ICUAW |
| Corner, Murray, & Brett, 2019; | No reported data for KRT and ICUAW |
| Cottereau et al., 2021; | No reported data for KRT and ICUAW |
| Cottini et al., 2013; | No reported data for KRT and ICUAW |
| Coursin, Updike, & Maki, 2000; | No reported data for KRT and ICUAW |
| Cozza, Do, Ganti, & Depa, 2021; | No reported data for KRT and ICUAW |
| Ctri, 2020; | No reported data for KRT and ICUAW |
| Curkovic et al., 2010; | No reported data for KRT and ICUAW |
| Da Silva et al., 2021; | No reported data for KRT and ICUAW |
| Dabar, Harmouche, Habr, Riachi, & Jaber, 2015; | No reported data for KRT and ICUAW |
| Dadras et al., 2019; | No reported data for KRT and ICUAW |
| Daher et al., 2008; | No reported data for KRT and ICUAW |
| Damak et al., 2011; | No reported data for KRT and ICUAW |
| Darvall et al., 2019; | No reported data for KRT and ICUAW |
| Daste et al., 2023; | No reported data for KRT and ICUAW |
| Davidson, Shaukat, Jenabzadeh, & Gupte, 2013; | No reported data for KRT and ICUAW |
| Davies et al., 2021; | No reported data for KRT and ICUAW |
| Davis et al., 2017; | No reported data for KRT and ICUAW |
| de Ávila et al., 2020; | No reported data for KRT and ICUAW |
| de Campos Biazon et al., 2021; | No reported data for KRT and ICUAW |
| de Carvalho, 2020; | No reported data for KRT and ICUAW |
| de Filette et al., 2022; | No reported data for KRT and ICUAW |
| de Gomes Figueiredo, Frazão, Werlang, Peltz, & Sobral Filho, 2023; | No reported data for KRT and ICUAW |
| de Sousa Arantes Ferreira et al., 2020; | No reported data for KRT and ICUAW |
| de Souza et al., 2008; | No reported data for KRT and ICUAW |
| Debella, Heckman, Venegas-Borsellino, & Baig, 2019; | No reported data for KRT and ICUAW |
| Dedy, Coghill, Chandrashekar, & Bindra, 2016; | No reported data for KRT and ICUAW |
| Dee, Thomas, & Gulbis, 2014; | No reported data for KRT and ICUAW |
| Deepa & Muralidhar, 2012; | No reported data for KRT and ICUAW |
| Delgado, Ruggeri, & Calvo, 2023; | No reported data for KRT and ICUAW |
| R. P. Dellinger et al., 2004; | No reported data for KRT and ICUAW |
| R. Phillip Dellinger et al., 2008; | No reported data for KRT and ICUAW |
| Demirjian et al., 2011; | No reported data for KRT and ICUAW |
| Demızız Gülmez, Akça, & Turan, 2022; | No reported data for KRT and ICUAW |
| Deng et al., 2023; | No reported data for KRT and ICUAW |
| Derespina et al., 2020; | No reported data for KRT and ICUAW |
| Dettling-Ihnenfeldt, Wieske, Horn, Nollet, & van der Schaaf, 2017; | No reported data for KRT and ICUAW |
| Dhaliwal, Zberea, Karthikeyan, & Singh, 2022; | No reported data for KRT and ICUAW |
| Dharod et al., 2013; | No reported data for KRT and ICUAW |
| Diaz Ballve et al., 2017; | No reported data for KRT and ICUAW |
| Dichtwald, Weinbroum, Sorkine, Ekstein, & Dahan, 2012; | No reported data for KRT and ICUAW |
| Dilken et al., 2020; | No reported data for KRT and ICUAW |
| Dimopoulos et al., 2020; | No reported data for KRT and ICUAW |
| Dimopoulos et al., 2023; | No reported data on KRT |
| Ding et al., 2022; | No reported data for KRT and ICUAW |
| Diniz, Dos Santos, Chagas, & Daher, 2021; | No reported data for KRT and ICUAW |
| Diószegi et al., 2018; | No reported data for KRT and ICUAW |
| Ditali et al., 2022; | No reported data for KRT and ICUAW |
| Dizman, Bahat, Özkanli, & Özkök, 2016; | No reported data for KRT and ICUAW |
| Djalali, Moore, & Kelly, 2005; | No reported data for KRT and ICUAW |
| Dłuski et al., 2020; | No reported data for KRT and ICUAW |
| Doig & Solverson, 2020; | No reported data for KRT and ICUAW |
| Dominedò, D’Avino, Martinotti, & Cingolani, 2021; | No reported data for KRT and ICUAW |
| J. Dong, Cao, Tanner, & Kundranda, 2020; | No reported data for KRT and ICUAW |
| S. B. Dong et al., 2021; | No reported data for KRT and ICUAW |
| Dongol, Sapkota, Devkota, Pandey, & Bhattarai, 2022; | No reported data for KRT and ICUAW |
| Donlon et al., 2021; | No reported data for KRT and ICUAW |
| Dorazio et al., 2023; | No reported data for KRT and ICUAW |
| dos Santos, da Silva Carvalho, Peres, Delfino, & Grion, 2020; | No reported data for KRT and ICUAW |
| Doyle, Meyer, Breen, & Hunt, 2020; | No reported data for KRT and ICUAW |
| Dravid et al., 2021; | No reported data for KRT and ICUAW |
| Dres et al., 2017; | No reported data for KRT and ICUAW |
| Dres et al., 2019; | A post hoc analysis and no reported data for KRT and ICUAW |
| Dresen, Weißbrich, Fimmers, Putensen, & Stehle, 2021; | No reported data for KRT and ICUAW |
| Drks, 2013; | No reported data for KRT and ICUAW |
| Du Bose et al., 2016; | No reported data for KRT and ICUAW |
| Du et al., 2022; | No reported data for KRT and ICUAW |
| Dua, Lavingia, Deslarzes-Dubuis, Dake, & Lee, 2019; | No reported data for KRT and ICUAW |
| Dubin et al., 2021; | No reported data for KRT and ICUAW |
| Duceau et al., 2022; | No reported data for KRT and ICUAW |
| Ducic et al., 2021; | No reported data for KRT and ICUAW |
| Dumkow, Voss, Peters, & Jennings, 2012; | No reported data for KRT and ICUAW |
| Dunphy, Singh, & Keating, 2017; | No reported data for KRT and ICUAW |
| Durazo et al., 2021; | No reported data for KRT and ICUAW |
| Duymun & Nasir, 2020; | No reported data for KRT and ICUAW |
| Dworschak et al., 2008; | No reported data for KRT and ICUAW |
| Eagan, Ramdharry, & Smailes, 2020; | No reported data for KRT and ICUAW |
| Ebbeson, De Kock, Penny, & Kollman, 2009; | No reported data for KRT and ICUAW |
| Ebdrup, Druey, & Mogensen, 2018; | No reported data for KRT and ICUAW |
| Eggmann et al., 2020; | No reported data for KRT and ICUAW |
| Egi et al., 2021; | No reported data for KRT and ICUAW |
| Eisenburger, Laggner, Lenz, & Druml, 2000; | No reported data for KRT and ICUAW |
| El-Abdellati et al., 2013; | No reported data for KRT and ICUAW |
| El-Sayed Ahmad et al., 2018; | No reported data for KRT and ICUAW |
| Elahi, Houng, Trahair, Ravindranathan, & Grant, 2012; | No reported data for KRT and ICUAW |
| Elías, Munro, & Liang, 2020; | No reported data for KRT and ICUAW |
| Elvia, Lestari, & George, 2021; | No reported data for KRT and ICUAW |
| Engelhardt et al., 2022a, 2022b; | No reported data for KRT and ICUAW |
| Ertuğlu, Kanbay, Afşar, Elsürer Afşar, & Kanbay, 2020; | No reported data for KRT and ICUAW |
| Espiritu et al., 2021; | No reported data for KRT and ICUAW |
| Esposito et al., 2017; | No reported data for KRT and ICUAW |
| Euctr, 2020; | No reported data for KRT and ICUAW |
| F. Andersen et al., 2017; | No reported data for KRT and ICUAW |
| Fadila & Wool, 2015; | No reported data for KRT and ICUAW |
| Fagoni et al., 2021; | No reported data for KRT and ICUAW |
| Farfán & Piérola, 2021; | No reported data for KRT and ICUAW |
| Fayed, Pivazyan, Conte, Chang, & Mai, 2020; | No reported data for KRT and ICUAW |
| Fearnley, Lines, Lewington, & Bodenham, 2011; | No reported data for KRT and ICUAW |
| Feghali et al., 2021; | No reported data for KRT and ICUAW |
| Feillet et al., 2003; | No reported data for KRT and ICUAW |
| Felli, Skhirtladze-Dworschak, Opfermann, & Dworschak, 2019; | No reported data for KRT and ICUAW |
| Felten-Barentsz et al., 2018; | No reported data for KRT and ICUAW |
| H. Feng et al., 2021; | No reported data for KRT and ICUAW |
| W. Feng et al., 2023; | No reported data for KRT and ICUAW |
| W. Feng et al., 2022; | No reported data for KRT and ICUAW |
| X. Feng et al., 2020; | No reported data for KRT and ICUAW |
| Z. Feng et al., 2021; | No reported data for KRT and ICUAW |
| Ferré et al., 2023; | No reported data for KRT and ICUAW |
| Fetterplace et al., 2019; | No reported data for KRT and ICUAW |
| Fiaccadori et al., 2021; | No reported data for KRT and ICUAW |
| Files et al., 2020; | No reported data for KRT and ICUAW |
| Fill et al., 2017; | No reported data for KRT and ICUAW |
| Firstiogusran, Yoshida, Hashimoto, Iwata, & Fujino, 2022; | No reported data for KRT and ICUAW |
| Fisse et al., 2021; | No reported data for KRT and ICUAW |
| Fitri & Aditianingsih, 2022; | No reported data for KRT and ICUAW |
| FitzMaurice, McCann, Walshaw, & Greenwood, 2021; | No reported data for KRT and ICUAW |
| Flatres et al., 2020; | No reported data for KRT and ICUAW |
| Flores & Checchia, 2017; | No reported data for KRT and ICUAW |
| Fluher et al., 2022; | No reported data for KRT and ICUAW |
| Folkestad et al., 2020; | No reported data for KRT and ICUAW |
| Fonseka et al., 2023; | No reported data for KRT and ICUAW |
| Fontela et al., 2021; | No reported data for KRT and ICUAW |
| Foti et al., 2020; | No reported data for KRT and ICUAW |
| França et al., 2020; | No reported data for KRT and ICUAW |
| Frawley, Powell, McQuiston, Gulvik, & Bégué, 2018; | No reported data for KRT and ICUAW |
| Freeman, Moir, Lowis, & Tam, 2021; | No reported data for KRT and ICUAW |
| Fröhlich, Lambe, & O'Dea, 2011; | No reported data for KRT and ICUAW |
| Frohlich, Ryan, & Fagan, 2011; | No reported data for KRT and ICUAW |
| Frydrychowicz et al., 2017; | No reported data for KRT and ICUAW |
| Fuest et al., 2023; | No reported data for KRT and ICUAW |
| Fujisawa, Miyanaga, Takeji, Shirota, & Ueda, 2023; | No reported data for KRT and ICUAW |
| Fujiwara et al., 2018; | No reported data for KRT and ICUAW |
| Gabriel, Noel, & Accoceberry, 2012; | No reported data for KRT and ICUAW |
| Gaddam, Velagapudi, Abu Sitta, & Kanzy, 2017; | No reported data for KRT and ICUAW |
| Galindo Martín, Monares Zepeda, & Lescas Méndez, 2017; | No reported data for KRT and ICUAW |
| Gallis, Kasprzak, Cucuruz, & Kopp, 2017; | No reported data for KRT and ICUAW |
| Galloway & Doughty, 2007; | No reported data for KRT and ICUAW |
| Gamakaranage et al., 2011; | No reported data for KRT and ICUAW |
| Game, Selby, & McIntyre, 2013; | No reported data for KRT and ICUAW |
| Gao et al., 2019; | No reported data for KRT and ICUAW |
| Gara et al., 2021; | No reported data for KRT and ICUAW |
| Garatti, Wu, Ammirati, & Sacco, 2022; | No reported data for KRT and ICUAW |
| Gardenier et al., 2016; | No reported data for KRT and ICUAW |
| Garros et al., 2017; | No reported data for KRT and ICUAW |
| Gatti et al., 2023; | No reported data for KRT and ICUAW |
| Gayathri, Bhargavi, Mani, & Sathyamurthy, 2023; | No reported data for KRT and ICUAW |
| Gede Yasa Asmara, Pebruanto, & Made Arya Winatha, 2022; | No reported data for KRT and ICUAW |
| Gee, Outsen, Becknell, & Schwaderer, 2015; | No reported data for KRT and ICUAW |
| Geeting, Alibrahim, Patel, Kumar, & Mallory, 2023; | No reported data for KRT and ICUAW |
| Gehri et al., 2024; | No reported data for KRT and ICUAW |
| Gentili et al., 2008; | No reported data for KRT and ICUAW |
| Georgiou, Krokidis, Elworthy, & Dimopoulos, 2016; | No reported data for KRT and ICUAW |
| Gerdts, Vloemans, & Kreis, 2007; | No reported data for KRT and ICUAW |
| Gergen, Madsen, Tilva, Smith, & Weyant, 2021; | No reported data for KRT and ICUAW |
| Gerstner, Rafalski, & Pankiewicz, 2022; | No reported data for KRT and ICUAW |
| Ghatak, Singh, & Baronia, 2012; | No reported data for KRT and ICUAW |
| N. Ghati et al., 2020; | No reported data for KRT and ICUAW |
| Nirmal Ghati et al., 2020; | No reported data for KRT and ICUAW |
| Ginsberg, Thurman, Scalea, & Stein, 2016; | No reported data for KRT and ICUAW |
| Giraldo et al., 2023; | No reported data for KRT and ICUAW |
| Giray et al., 2023; | No reported data for KRT and ICUAW |
| Girgin et al., 2017; | No reported data for KRT and ICUAW |
| Giri, Akhtar, Laha, & Sinha, 2021; | No reported data for KRT and ICUAW |
| Giuliani et al., 2013; | No reported data for KRT and ICUAW |
| Giuliano et al., 2021; | No reported data for KRT and ICUAW |
| Gkiourtzis, Tramma, Papadopoulou-Legbelou, Moutafi, & Evangeliou, 2023; | No reported data for KRT and ICUAW |
| Gleeson, Noori, Lightstone, & Webster, 2021; | No reported data for KRT and ICUAW |
| Godo et al., 2017; | No reported data for KRT and ICUAW |
| Gombert et al., 2022; | No reported data for KRT and ICUAW |
| Gombert et al., 2021; | No reported data for KRT and ICUAW |
| C. L. R. Gomes, Yamane, Ruzany, & Rocco Suassuna, 2022; | No reported data for KRT and ICUAW |
| R. C. Gomes et al., 2013; | No reported data for KRT and ICUAW |
| Gong, Long, Xu, Yang, & Guo, 2018; | No reported data for KRT and ICUAW |
| Gonzalez Reyes, Perez Del Nogal, Sierra David, & Bastidas Palacios, 2023; | No reported data for KRT and ICUAW |
| Gopalakrishnan et al., 2020; | No reported data for KRT and ICUAW |
| Gordon, Ferris, & Pauli, 2023; | No reported data for KRT and ICUAW |
| Goubella et al., 2017; | No reported data for KRT and ICUAW |
| Gouin et al., 2021; | No reported data for KRT and ICUAW |
| Goyal, Dashey, Zlocha, & HannaJumma, 2020; | No reported data for KRT and ICUAW |
| Gräfe et al., 2023; | No reported data for KRT and ICUAW |
| Grancini et al., 2022; | No reported data for KRT and ICUAW |
| Grasselli et al., 2008; | No reported data for KRT and ICUAW |
| Gravos et al., 2018; | No reported data for KRT and ICUAW |
| Green et al., 2022; | No reported data for KRT and ICUAW |
| Greene, Adams, Rogers, Berard-Collins, & Lorenzo, 2020; | No reported data for KRT and ICUAW |
| Grieb et al., 2021; | No reported data for KRT and ICUAW |
| Groves et al., 2014; | No reported data for KRT and ICUAW |
| Gruber et al., 2023; | No reported data for KRT and ICUAW |
| Grunow et al., 2023; | No reported data for KRT and ICUAW |
| Grunow et al., 2019; | No reported data for KRT and ICUAW |
| Grunow et al., 2022; | No reported data for KRT and ICUAW |
| Grunow & Weber-Carstens, 2023; | No reported data for KRT and ICUAW |
| Guery et al., 2013; | No reported data for KRT and ICUAW |
| Guidet, de Lange, & Flaatten, 2018; | No reported data for KRT and ICUAW |
| Guinot et al., 2023; | No reported data for KRT and ICUAW |
| Gulati et al., 2014; | No reported data for KRT and ICUAW |
| Guliani et al., 2021; | No reported data for KRT and ICUAW |
| Gunal et al., 2004; | No reported data for KRT and ICUAW |
| Gunasekaran, Mathew, Sudarsan, & Iyyadurai, 2019; | No reported data for KRT and ICUAW |
| Guntz, Layios, & Damas, 2014; | No reported data for KRT and ICUAW |
| Guo, Lin, & Lin, 2022; | No reported data for KRT and ICUAW |
| P. Gupta et al., 2020; | No reported data for KRT and ICUAW |
| P. Gupta & Verma, 2017; | No reported data for KRT and ICUAW |
| S. Gupta, Prabhu, Gupta, & Niblett, 1998; | No reported data for KRT and ICUAW |
| Gurjar et al., 2011; | No reported data for KRT and ICUAW |
| Gwinner et al., 2007; | No reported data for KRT and ICUAW |
| Habr et al., 2020; | No reported data for KRT and ICUAW |
| Haeberle et al., 2020; | No reported data for KRT and ICUAW |
| Haglin, 2016; | No reported data for KRT and ICUAW |
| Hahn et al., 2020; | No reported data for KRT and ICUAW |
| Haines et al., 2019; | No reported data for KRT and ICUAW |
| Hajsadeghi, Gholizadeh Mesgarha, Pour Mohammad, Saberi Shahrbabaki, & Talebi, 2022; | No reported data for KRT and ICUAW |
| Hamadeh et al., 2021; | No reported data for KRT and ICUAW |
| Hammoud, Fulmer, Hamner, & El Atrouni, 2023; | No reported data for KRT and ICUAW |
| Hampton et al., 2022; | No reported data for KRT and ICUAW |
| Han, You, & Jung, 2022; | No reported data for KRT and ICUAW |
| Hannemann et al., 2022; | No reported data for KRT and ICUAW |
| Harky et al., 2021; | No reported data for KRT and ICUAW |
| Harrois et al., 2018; | No reported data for KRT and ICUAW |
| Harvey & Stanton, 2017; | No reported data for KRT and ICUAW |
| Hasan et al., 2020; | No reported data for KRT and ICUAW |
| Hayes, Holland, Pellegrino, Mathur, & Hodgson, 2018; | No reported data for KRT and ICUAW |
| Heath & Courtright, 2017; | No reported data for KRT and ICUAW |
| Heggemont et al., 2017; | No reported data for KRT and ICUAW |
| Heidarpour et al., 2021; | No reported data for KRT and ICUAW |
| Heise, Skolnik, Raschke, Owen-Reece, & Graeme, 2016; | No reported data for KRT and ICUAW |
| Heister, Bohnert, Heyne, Birkenfeld, & Artunc, 2023; | No reported data for KRT and ICUAW |
| Heneghan et al., 2014; | No reported data for KRT and ICUAW |
| Herbst, Helleberg, Overgaard-Steensen, & Holmgaard, 2023; | No reported data for KRT and ICUAW |
| Hermanns-Clausen, Sydow, & Desel, 2005; | No reported data for KRT and ICUAW |
| Hermans et al., 2007; | Data included in study Weber-Carstens 2009 |
| Hermans et al., 2013; | No reported data on KRT |
| Hermine et al., 2021; | No reported data for KRT and ICUAW |
| Hernández-Socorro, Saavedra, López-Fernández, Lübbe-Vazquez, & Ruiz-Santana, 2021; | No reported data for KRT and ICUAW |
| Heyland et al., 2023; | No reported data for KRT and ICUAW |
| Hierholzer, Majdinasab, Young, & Kauffman, 2023; | No reported data for KRT and ICUAW |
| F. J. Hill, McCloskey, & Sheerin, 2015; | No reported data for KRT and ICUAW |
| K. L. Hill, Wu, Kusumoto, Sherman, & Savoie, 2020; | No reported data for KRT and ICUAW |
| Hin et al., 2012; | No reported data for KRT and ICUAW |
| Hirose et al., 2022; | No reported data for KRT and ICUAW |
| Ho, Yang, Wong, & Chong, 2018; | No reported data for KRT and ICUAW |
| Hodgson et al., 2022; | No reported data for KRT and ICUAW |
| Hodgson & Tipping, 2017; | No reported data for KRT and ICUAW |
| Hofmann, Waldherr, & Schwenger, 2005; | No reported data for KRT and ICUAW |
| Höhn & Speelberg, 2014; | No reported data for KRT and ICUAW |
| Holmberg, Ahn, & Peterzén, 2017; | No reported data for KRT and ICUAW |
| Horiuchi, Uwatoko, & Houzen, 2015; | No reported data for KRT and ICUAW |
| Horn & Hermans, 2017; | No reported data for KRT and ICUAW |
| Horoz et al., 2008; | No reported data for KRT and ICUAW |
| Hosoya et al., 1997; | No reported data for KRT and ICUAW |
| Hoste & Kellum, 2006; | No reported data for KRT and ICUAW |
| Hough et al., 2015; | No reported data for KRT and ICUAW |
| Howell, Brundige, & Langworthy, 2007; | No reported data for KRT and ICUAW |
| Hsiao et al., 2016; | No reported data for KRT and ICUAW |
| Y. Hu, Hu, Xiao, & Li, 2019; | No reported data for KRT and ICUAW |
| Z. Hu, Muller, Slone, & Inaba, 2024; | No reported data for KRT and ICUAW |
| C. E. Huang & Huang, 2010; | No reported data for KRT and ICUAW |
| C. Y. Huang, Tsai, Wu, Chen, & Wang, 2022; | No reported data for KRT and ICUAW |
| D. Huang et al., 2020; | No reported data for KRT and ICUAW |
| D. Huang et al., 2023; | No reported data for KRT and ICUAW |
| H. Huang, Huang, Chen, & Okamoto, 2024; | No reported data for KRT and ICUAW |
| L. Huang, Shi, Li, & Li, 2021; | No reported data for KRT and ICUAW |
| S. W. Huang sw., 2022; | No reported data for KRT and ICUAW |
| T. T. M. Huang et al., 2021; | No reported data for KRT and ICUAW |
| W. C. Huang et al., 2022; | No reported data for KRT and ICUAW |
| Y. Q. Huang, Tremblay, Chapdelaine, Luong, & Carrier, 2020; | No reported data for KRT and ICUAW |
| Huckriede et al., 2021; | No reported data for KRT and ICUAW |
| Hui et al., 2022; | No reported data for KRT and ICUAW |
| Hui, Lam, Cheung, & Ku, 2023; | No reported data for KRT and ICUAW |
| Huisa, Chapin, & Adair, 2009; | No reported data for KRT and ICUAW |
| Hulse, 2021; | No reported data for KRT and ICUAW |
| Hummel, Glotzbach, Menon, Griffiths, & Lal, 2020; | No reported data for KRT and ICUAW |
| Hunt et al., 2021; | No reported data for KRT and ICUAW |
| Hunter et al., 2017; | No reported data for KRT and ICUAW |
| Hussain, Hall, Depczynski, & Connor, 2014; | No reported data for KRT and ICUAW |
| Ibrahim et al., 2021; | No reported data for KRT and ICUAW |
| Ibrahimi et al., 2014; | No reported data for KRT and ICUAW |
| Iftikhar, Dar, & Haw, 2022; | No reported data for KRT and ICUAW |
| Inan et al., 2022; | No reported data for KRT and ICUAW |
| Inoue et al., 2007; | No reported data for KRT and ICUAW |
| Intiso, 2018; | No reported data for KRT and ICUAW |
| Intiso, Centra, Amoruso, Gravina, & Rienzo, 2022; | No reported data for KRT and ICUAW |
| Isfort, McVerry, Shutter, Kim, & Lacomis, 2022; | No reported data for KRT and ICUAW |
| Isoardi et al., 2023; | No reported data for KRT and ICUAW |
| Ito et al., 2020; | No reported data for KRT and ICUAW |
| Iwasa et al., 2014; | No reported data for KRT and ICUAW |
| Iyoda et al., 2003; | No reported data for KRT and ICUAW |
| Jabur, Nasa, Mohammed, Kulkarni, & Tomaraei, 2018; | No reported data for KRT and ICUAW |
| Jadhav, Vijaya, Alexis, & Pandit, 2018; | No reported data for KRT and ICUAW |
| Jahns, Pineau Mitchell, & Auzinger, 2018; | No reported data for KRT and ICUAW |
| Jain, Nand, Giri, & Bhutani, 2019; | No reported data for KRT and ICUAW |
| Jamuar et al., 2012; | No reported data for KRT and ICUAW |
| Jana, Janga, Greenberg, & Gulati, 2021; | No reported data for KRT and ICUAW |
| Jang, Yoon, Ahn, Lee, & Shin, 2018; | No reported data for KRT and ICUAW |
| Jaquet et al., 2022; | No reported data for KRT and ICUAW |
| Jayasuriya & Movahed, 2009; | No reported data for KRT and ICUAW |
| Jeong, Kim, Kim, & Kim, 2018; | No reported data for KRT and ICUAW |
| Jerman, Andonova, Persic, & Gubensek, 2022; | No reported data for KRT and ICUAW |
| Jha & Shutter, 2017; | No reported data for KRT and ICUAW |
| Jhang et al., 2014; | No reported data for KRT and ICUAW |
| Ji & Won, 2023; | No reported data for KRT and ICUAW |
| Jiang et al., 2021; | No reported data for KRT and ICUAW |
| J. Jiao et al., 2017; | No reported data for KRT and ICUAW |
| R. Jiao et al., 2022; | No reported data for KRT and ICUAW |
| Jin, Jeswant Dillon, Tjen Jhung, & Isman Rusani, 2020; | No reported data for KRT and ICUAW |
| Jiroutková et al., 2017; | No reported data for KRT and ICUAW |
| J. F. John et al., 2018; | No reported data for KRT and ICUAW |
| S. G. John, William, Murugapandian, & Thajudeen, 2014; | No reported data for KRT and ICUAW |
| Jongkind, Kievit, & Wiersema, 2016; | No reported data for KRT and ICUAW |
| Jonsdottir et al., 2022; | No reported data for KRT and ICUAW |
| Jubina et al., 2023; | No reported data for KRT and ICUAW |
| Jutras et al., 2018; | No reported data for KRT and ICUAW |
| Kade, Osman, Antosiewicz, & Wańkowicz, 2012; | No reported data for KRT and ICUAW |
| Kahraman et al., 2022; | No reported data for KRT and ICUAW |
| Kainz, Bsuchner, Schellongowski, & Dworschak, 2021; | No reported data for KRT and ICUAW |
| Kala & Abudayyeh, 2017; | No reported data for KRT and ICUAW |
| Kallel et al., 2005; | No reported data for KRT and ICUAW |
| Kam & Cardone, 2007; | No reported data for KRT and ICUAW |
| Kamada, Suzuki, Yamamoto, Nomura, & Kushimoto, 2017; | No reported data for KRT and ICUAW |
| Kanagasundaram & Arunachalam, 2015; | No reported data for KRT and ICUAW |
| Kantola, Kantola, Koivusalo, Höckerstedt, & Isoniemi, 2009; | No reported data for KRT and ICUAW |
| Karaaslan et al., 2021; | No reported data for KRT and ICUAW |
| Kasaoka et al., 2010; | No reported data for KRT and ICUAW |
| Kasinathan et al., 2021; | No reported data for KRT and ICUAW |
| Kasturiarachi, Robinson, Karkoska, & Gollamudi, 2024; | No reported data for KRT and ICUAW |
| Kasugai, Tajima, Jingushi, Uenishi, & Hirakawa, 2020; | No reported data for KRT and ICUAW |
| Katsuhara et al., 2015; | No reported data for KRT and ICUAW |
| Kaushik et al., 2012; | No reported data for KRT and ICUAW |
| Kayim Yildiz, Yildiz, Avci, Hasbek, & Kanat, 2021; | No reported data for KRT and ICUAW |
| Keenswijk, Dhont, Raes, Bael, & Vande Walle, 2018; | No reported data for KRT and ICUAW |
| Kemp et al., 2020; | No reported data for KRT and ICUAW |
| Kendirli et al., 2009; | No reported data for KRT and ICUAW |
| Keskin et al., 2015; | No reported data for KRT and ICUAW |
| Keyzer, Hoorens, Van Dorpe, & Bogaert, 2021; | No reported data for KRT and ICUAW |
| Khalid et al., 2022; | Cross-Sectional study |
| Khalil et al., 2023; | Cross-Sectional study |
| Khan, Maheshwari, & Haque, 2013; | No reported data for KRT and ICUAW |
| A. K. Khanna et al., 2023; | No reported data for KRT and ICUAW |
| S. Khanna et al., 2000; | No reported data for KRT and ICUAW |
| Kharibam, Jithesh, Kumar, & Pathania, 2021; | No reported data for KRT and ICUAW |
| Khosravi, Babaei, Azizi, & Samaee, 2021; | No reported data for KRT and ICUAW |
| Khoury, Mickey, Marrocco, & Katyal, 2018; | No reported data for KRT and ICUAW |
| Kihira et al., 2020; | No reported data for KRT and ICUAW |
| Kilit, Kilit, & Erarslan, 2017; | No reported data for KRT and ICUAW |
| D. Kim et al., 2020; | No reported data for KRT and ICUAW |
| H. W. Kim et al., 2022; | No reported data for KRT and ICUAW |
| J. E. Kim et al., 2017; | No reported data for KRT and ICUAW |
| J. H. Kim et al., 2018; | No reported data for KRT and ICUAW |
| M. G. Kim mg., 2017; | No reported data for KRT and ICUAW |
| S. H. Kim, Shin, Shin, & Jang, 2022; | No reported data for KRT and ICUAW |
| S. H. Kim, Song, & Jung, 2019; | No reported data for KRT and ICUAW |
| Kimball & Yost, 2011; | No reported data for KRT and ICUAW |
| Kinoshita et al., 2021; | No reported data for KRT and ICUAW |
| Klawitter, Oppitz, et al., 2022; | No reported data for KRT and ICUAW |
| Klawitter, Schaller, Söhle, Reuter, & Ehler, 2022; | No reported data for KRT and ICUAW |
| Klawitter, Walter, et al., 2022; | No reported data for KRT and ICUAW |
| Kobara, Rad, Grunwell, Coopersmith, & Kamaleswaran, 2022; | No reported data for KRT and ICUAW |
| Koda et al., 2013; | No reported data for KRT and ICUAW |
| Kodadek et al., 2022; | No reported data for KRT and ICUAW |
| Kodati et al., 2022; | No reported data for KRT and ICUAW |
| Kofler et al., 2023; | No reported data for KRT and ICUAW |
| Kok, Jitta, Veneman, & Veneman, 2019; | No reported data for KRT and ICUAW |
| Kondo et al., 2006; | No reported data for KRT and ICUAW |
| Koppel, Schirop, Barckow, & Frei, 1995; | No reported data for KRT and ICUAW |
| Korotchaeva et al., 2022; | No reported data for KRT and ICUAW |
| Köseoğlu Toksoy et al., 2022 | No reported data for KRT and ICUAW |
| Kotton, Soboh, & Bisharat, 2015; | No reported data for KRT and ICUAW |
| Kourek et al., 2024; | No reported data for KRT and ICUAW |
| Koyama et al., 2015; | No reported data for KRT and ICUAW |
| Kozik & Wikerd, 2023; | No reported data for KRT and ICUAW |
| Kraft et al., 2015; | No reported data for KRT and ICUAW |
| Kramer et al., 2006; | No reported data for KRT and ICUAW |
| Krishna Kumar et al., 2022; | No reported data for KRT and ICUAW |
| Krol et al., 2021; | No reported data for KRT and ICUAW |
| Krupesh et al., 2021; | No reported data for KRT and ICUAW |
| Kubik, Smyła, Herba, Białka, & Rydel, 2022; | No reported data for KRT and ICUAW |
| Kularatne et al., 2014; | No reported data for KRT and ICUAW |
| Kumthekar, Shukla, & Purandare, 2023; | No reported data for KRT and ICUAW |
| Kundu, Almasri, Moza, Ghose, & Assaly, 2013; | No reported data for KRT and ICUAW |
| Kuok & Chan, 2021; | No reported data for KRT and ICUAW |
| Kutleša, Lepur, Bukovski, Lepur, & Baršić, 2009; | No reported data for KRT and ICUAW |
| Kypreos & Mehta, 2023; | No reported data for KRT and ICUAW |
| Lachmann et al., 2020; | No reported data for KRT and ICUAW |
| Lagi, Corti, Meli, Pinto, & Bartoloni, 2013; | No reported data for KRT and ICUAW |
| Lal, Nabzdyk, Ramakrishna, & Radosevich, 2020; | No reported data for KRT and ICUAW |
| Lamou et al., 2014; | No reported data for KRT and ICUAW |
| Langley, Zeicu, Whitton, & Pauls, 2020; | No reported data for KRT and ICUAW |
| Langrand et al., 2013; | No reported data for KRT and ICUAW |
| Larzon & Mathisen, 2010; | No reported data for KRT and ICUAW |
| Laurence et al., 2020; | No reported data for KRT and ICUAW |
| Le et al., 2021; | No reported data for KRT and ICUAW |
| Lechner et al., 2017; | No reported data for KRT and ICUAW |
| Lecluyse, Couture, & Denault, 2017; | No reported data for KRT and ICUAW |
| C. S. Lee, Hsiao, Hung, Hsu, & Chang, 2017; | No reported data for KRT and ICUAW |
| G. Y. Lee et al., 2022; | No reported data for KRT and ICUAW |
| J. H. Lee et al., 2021; | No reported data for KRT and ICUAW |
| Legris et al., 2014; | No reported data for KRT and ICUAW |
| Lehner, Schöpf, Harler, Pechlaner, & Joannidis, 2014; | No reported data for KRT and ICUAW |
| Lehr et al., 2018; | No reported data for KRT and ICUAW |
| Leijten et al., 1996; | No reported data for KRT and ICUAW |
| Leonhardt et al., 2023; | No reported data for KRT and ICUAW |
| Lerma et al., 2017; | No reported data for KRT and ICUAW |
| Lessel et al., 2020; | No reported data for KRT and ICUAW |
| Leventoğlu et al., 2022; | No reported data for KRT and ICUAW |
| Lévesque, Millaire, Corsilli, Rioux-Massé, & Carrier, 2020; | No reported data for KRT and ICUAW |
| Levine & Vijayan, 2023; | No reported data for KRT and ICUAW |
| A. P. Z. Li, Thomas, Gokmen, & Kariyawasam, 2021; | No reported data for KRT and ICUAW |
| C. Li, Zhang, & Zhu, 2021; | No reported data for KRT and ICUAW |
| D. S. Li et al., 2024; | No reported data for KRT and ICUAW |
| L. Li, Bai, Zhang, & Sun, 2022; | No reported data for KRT and ICUAW |
| L. Li & Liu, 2021; | No reported data for KRT and ICUAW |
| M. Li et al., 2021; | No reported data for KRT and ICUAW |
| Q. Li et al., 2020; | No reported data for KRT and ICUAW |
| Q. Li, Hu, Kang, & Zhou, 2021; | No reported data for KRT and ICUAW |
| R. Y. Li et al., 2020; | No reported data for KRT and ICUAW |
| W. Li, Gong, Wu, & Liu, 2014; | No reported data for KRT and ICUAW |
| X. Li et al., 2022; | No reported data for KRT and ICUAW |
| X. Li, Lin, & Mu, 2020; | No reported data for KRT and ICUAW |
| X. Li et al., 2023; | No reported data for KRT and ICUAW |
| Y. Li, N. Dörmann, et al., 2023; | No reported data for KRT and ICUAW |
| Y. Li, Ji, Jing, Huang, & Duan, 2021; | No reported data for KRT and ICUAW |
| Y. Li, Xi, Jiang, & Zhu, 2019; | No reported data for KRT and ICUAW |
| Y. Li, Xie, Li, & Lu, 2023; | No reported data for KRT and ICUAW |
| Lian et al., 2020; | No reported data for KRT and ICUAW |
| J. H. B. Lim, Robinson, & Savige, 2023; | No reported data for KRT and ICUAW |
| J. Y. Lim & Lee, 2023; | No reported data for KRT and ICUAW |
| K. K. Lim, Kaye, & Drummond, 2009; | No reported data for KRT and ICUAW |
| C. Lin et al., 2023; | No reported data for KRT and ICUAW |
| G. Lin, Lin, & Liao, 2020; | No reported data for KRT and ICUAW |
| J. J. Lin, McKenney, Price, Morrison, & Novotny, 2002; | No reported data for KRT and ICUAW |
| T. K. Lin et al., 2024; | No reported data for KRT and ICUAW |
| Y. Lin et al., 2023; | No reported data for KRT and ICUAW |
| Y. H. Lin, 2020; | No reported data for KRT and ICUAW |
| Ling, Fong, & Chan, 2023; | No reported data for KRT and ICUAW |
| Linko, Laukkanen, Koljonen, Rapola, & Varpula, 2014; | No reported data for KRT and ICUAW |
| B. Liu, He, & Long, 2021; | No reported data for KRT and ICUAW |
| C. Liu et al., 2021; | No reported data for KRT and ICUAW |
| F. Liu et al., 2009; | No reported data for KRT and ICUAW |
| P. Liu et al., 2021; | No reported data for KRT and ICUAW |
| Y. Liu et al., 2021; | No reported data for KRT and ICUAW |
| Y. Liu et al., 2020; | No reported data for KRT and ICUAW |
| Lizwan, Lee, Chong, & Chua, 2023; | No reported data for KRT and ICUAW |
| Lobo et al., 2020; | No reported data for KRT and ICUAW |
| Lobo, Lokhande, Chakurkar, & D'Costa, 2021; | No reported data for KRT and ICUAW |
| Lochter, Sommer, Moerer, & Stephani, 2023; | No reported data for KRT and ICUAW |
| Lodhi, Akcan, Mojarrab, Sajjad, & Blonsky, 2021; | No reported data for KRT and ICUAW |
| Loftus, Moore, & Moldawer, 2017; | No reported data for KRT and ICUAW |
| Lombi, Muryan, Canzonieri, & Trimarchi, 2016; | No reported data for KRT and ICUAW |
| Luan et al., 2023; | No reported data for KRT and ICUAW |
| Lubana, Genin, Singh, & De La Cruz, 2015; | No reported data for KRT and ICUAW |
| Lucaj et al., 2022; | No reported data for KRT and ICUAW |
| Luckoor, Salehi, & Kunadu, 2017; | No reported data for KRT and ICUAW |
| Lugthart et al., 2024; | No reported data for KRT and ICUAW |
| Lust, Gong, Remiker, & Rossoff, 2021; | No reported data for KRT and ICUAW |
| H. Ma, Bavishi, & Jain, 2023; | No reported data for KRT and ICUAW |
| W. Ma, Suhitharan, Shah, & Kothandan, 2021; | No reported data for KRT and ICUAW |
| Maan et al., 2022; | No reported data for KRT and ICUAW |
| Mader, Lührs, Herget-Rosenthal, & Langenbeck, 2019; | No reported data for KRT and ICUAW |
| Mahran et al., 2023; | No reported data for KRT and ICUAW |
| Manabe, Yanagi, Ozawa, & Takagi, 2019; | No reported data for KRT and ICUAW |
| Mand, Donath, Leonhardt, Weber, & Kömhoff, 2023; | No reported data for KRT and ICUAW |
| Mannerkorpi, Raatiniemi, Kaikkonen, & Kaakinen, 2020; | No reported data for KRT and ICUAW |
| Mansour et al., 2023; | No reported data for KRT and ICUAW |
| Marahrens, Amann, Asmus, Erfurt, & Patschan, 2021; | No reported data for KRT and ICUAW |
| Maramattom, 2022; | No reported data for KRT and ICUAW |
| Marco, González-Muñoz, & Doti, 2023; | No reported data for KRT and ICUAW |
| Mariano et al., 2023; | No reported data for KRT and ICUAW |
| Marrero et al., 2020; | No reported data for KRT and ICUAW |
| Mart, Pun, Pandharipande, Jackson, & Ely, 2021; | No reported data for KRT and ICUAW |
| Martí-Pastor et al., 2023; | No reported data for KRT and ICUAW |
| Martin-Loeches et al., 2021; | No reported data for KRT and ICUAW |
| Martin-Loeches et al., 2011; | No reported data for KRT and ICUAW |
| K. Martin, Daining, & Gonzales, 2012; | No reported data for KRT and ICUAW |
| L. Martin, Ernst, Winter, Droege, & Mueller, 2016; | No reported data for KRT and ICUAW |
| Martins-Baltar et al., 2022; | No reported data for KRT and ICUAW |
| Marupudi et al., 2024; | No reported data for KRT and ICUAW |
| Mas-Font et al., 2017; | No reported data for KRT and ICUAW |
| Masolitin et al., 2022; | No reported data for KRT and ICUAW |
| Matsushima et al., 2021; | No reported data for KRT and ICUAW |
| Mayer et al., 2022; | No reported data for KRT and ICUAW |
| Mayer et al., 2020; | No reported data for KRT and ICUAW |
| Mayer et al., 2021; | No reported data for KRT and ICUAW |
| Mazza et al., 2020; | No reported data for KRT and ICUAW |
| Mbonde et al., 2023; | No reported data for KRT and ICUAW |
| McBeth, Dunham, Ball, & Kirkpatrick, 2012; | No reported data for KRT and ICUAW |
| McCann, Hunter, & McCann, 2002; | No reported data for KRT and ICUAW |
| McKenzie, Quinones, Mentzer, & Kruse, 2017; | No reported data for KRT and ICUAW |
| McPeake, Iwashyna, Devine, Mactavish, & Quasim, 2017; | No reported data for KRT and ICUAW |
| McWilliams, Atkins, Hodson, & Snelson, 2017; | No reported data for KRT and ICUAW |
| Medrinal et al., 2021; | No reported data for KRT and ICUAW |
| Méhes et al., 2012; | No reported data for KRT and ICUAW |
| Mehmood, Jaffar, Nazim, & Khasawneh, 2014; | No reported data for KRT and ICUAW |
| Mehra et al., 2021; | No reported data for KRT and ICUAW |
| Mehrpour, Saeedi, Hadianfar, Mégarbane, & Hoyte, 2021; | No reported data for KRT and ICUAW |
| Mehta et al., 2013; | No reported data for KRT and ICUAW |
| Melissa, Adit, Junaid, & Sean, 2022; | No reported data for KRT and ICUAW |
| Mendonca, Tamas, Kiraly, Talo, & Rajah, 2016; | No reported data for KRT and ICUAW |
| Meraz-Muñoz, Gomez-Ruiz, Correa-Rotter, & Ramirez-Sandoval, 2018; | No reported data for KRT and ICUAW |
| Merrill et al., 2023; | No reported data for KRT and ICUAW |
| Metterlein et al., 2011; | No reported data for KRT and ICUAW |
| Metzler et al., 2022; | No reported data for KRT and ICUAW |
| Meyer-Frießem et al., 2021; | No reported data for KRT and ICUAW |
| Meyer, Alt-Epping, Moerer, & Büttner, 2021; | No reported data for KRT and ICUAW |
| Miarons et al., 2021; | No reported data for KRT and ICUAW |
| Michail et al., 2013; | No reported data for KRT and ICUAW |
| Mijatovic, Blagaic, & Zupan, 2014; | No reported data for KRT and ICUAW |
| Mikkelsen & Toft, 2005; | No reported data for KRT and ICUAW |
| Miller-Smith, Flint, & Allen, 2021; | No reported data for KRT and ICUAW |
| Minami et al., 2023; | No reported data for KRT and ICUAW |
| Miranda, Lima, & Dourado, 2022; | No reported data for KRT and ICUAW |
| Mishra, Nugent, Dar, & Lado-Abeal, 2012; | No reported data for KRT and ICUAW |
| Mitobe et al., 2019; | No reported data for KRT and ICUAW |
| Mitsui et al., 2024; | No reported data for KRT and ICUAW |
| Miyabayashi et al., 2002; | No reported data for KRT and ICUAW |
| Miyara et al., 2021; | No reported data for KRT and ICUAW |
| Mizuguchi et al., 2004; | No reported data for KRT and ICUAW |
| Mocan, Szabo, Constantinescu, Cucoreanu, & Chira, 2022; | No reported data for KRT and ICUAW |
| Mohamed et al., 2021; | No reported data for KRT and ICUAW |
| H. A. E. M. Mohammed et al., 2023; | No reported data for KRT and ICUAW |
| I. Mohammed & Hussain, 2004; | No reported data for KRT and ICUAW |
| Mohd et al., 2018; | No reported data for KRT and ICUAW |
| Möhn et al., 2022; | No reported data for KRT and ICUAW |
| Monnier et al., 2020; | No reported data for KRT and ICUAW |
| Moon, Chun, & Cho, 2023; | No reported data for KRT and ICUAW |
| Moonen, Strookappe, & van Zanten, 2022; | No reported data for KRT and ICUAW |
| Morandi et al., 2017; | No reported data for KRT and ICUAW |
| Morelle, Kanaan, & Hantson, 2010; | No reported data for KRT and ICUAW |
| Moresco, Rugg, Ströhle, & Thoma, 2022; | No reported data for KRT and ICUAW |
| Mörgeli et al., 2021; | No reported data for KRT and ICUAW |
| Mori, Geirsson, Vallabhajosyula, & Assi, 2020; | No reported data for KRT and ICUAW |
| Morimoto, Sekino, Eishi, & Kozu, 2018; | No reported data for KRT and ICUAW |
| C. Morris, Gray, & Giovannelli, 2015; | No reported data for KRT and ICUAW |
| D. Morris et al., 2021; | No reported data for KRT and ICUAW |
| Mottard, Boulay, & Hautin, 2021; | No reported data for KRT and ICUAW |
| Mousavi et al., 2022; | No reported data for KRT and ICUAW |
| Muaddi, Ledgerwood, Sheridan, Dumont, & Nashar, 2022; | No reported data for KRT and ICUAW |
| Mugwagwa, Fischer, & Zailan, 2016; | No reported data for KRT and ICUAW |
| Mukpradab, Cussen, Ranse, Songwathana, & Marshall, 2023; | No reported data for KRT and ICUAW |
| Muñoz-Martínez et al., 2015; | No reported data for KRT and ICUAW |
| Murphy et al., 2018; | No reported data for KRT and ICUAW |
| Murugappan et al., 2019; | No reported data for KRT and ICUAW |
| Mustonen & Vuola, 2008; | No reported data for KRT and ICUAW |
| Nagatomi, Wakatake, Masui, Hayashi, & Fujitani, 2023; | No reported data for KRT and ICUAW |
| Nahrir, Sinha, & Siddiqui, 2012; | No reported data for KRT and ICUAW |
| Nakada et al., 2022; | No reported data for KRT and ICUAW |
| Nakamura et al., 2022; | No reported data for KRT and ICUAW |
| Nakamura et al., 2019; | No reported data for KRT and ICUAW |
| Nakanishi, Doi, Kawahara, Shiraishi, & Oto, 2021; | No reported data for KRT and ICUAW |
| Nakanishi et al., 2020; | No reported data for KRT and ICUAW |
| Nakano et al., 2021; | No reported data for KRT and ICUAW |
| Nakayama et al., 2021; | No reported data for KRT and ICUAW |
| Nalçacıoğlu et al., 2023; | No reported data for KRT and ICUAW |
| Nalesso et al., 2021; | No reported data for KRT and ICUAW |
| Naoi et al., 2022; | No reported data for KRT and ICUAW |
| Nardelli, Powers, Cope, & Rich, 2017; | No reported data for KRT and ICUAW |
| Nasello et al., 2023; | No reported data for KRT and ICUAW |
| Naser, Curfman, Marley, & Duke, 2022; | No reported data for KRT and ICUAW |
| Nassikovker, Holla, van der Hoeven, & Heunks, 2012; | No reported data for KRT and ICUAW |
| Navarro et al., 2021; | No reported data for KRT and ICUAW |
| Neary & Crabb, 2000; | No reported data for KRT and ICUAW |
| Needham et al., 2014; | Post-hospital outcome study and no reported data on KRT |
| Needham et al., 2021; | No reported data for KRT and ICUAW |
| Neoh, Tang, Looi, & Anita, 2020; | No reported data for KRT and ICUAW |
| Neto et al., 2024; | No reported data for KRT and ICUAW |
| Ney, Moll, & Kimball, 2022; | No reported data for KRT and ICUAW |
| Ni, Zhou, & Zhang, 2020; | No reported data for KRT and ICUAW |
| M. Nickels, Aitken, Walsham, Watson, & McPhail, 2017; | No reported data for KRT and ICUAW |
| M. R. Nickels, Aitken, Walsham, Barnett, & McPhail, 2017; | No reported data for KRT and ICUAW |
| Nielsen, Ingeholm, Holck, & Talbot, 2007; | No reported data for KRT and ICUAW |
| Nieto-Ríos, Vega-Miranda, & Serna-Higuita, 2016; | No reported data for KRT and ICUAW |
| Nieuwstraten, Statius van Eps, Wever, & Veger, 2023; | No reported data for KRT and ICUAW |
| Niibe et al., 2023; | No reported data for KRT and ICUAW |
| Nishida & Yoshii, 2023; | No reported data for KRT and ICUAW |
| Nlandu et al., 2022; | No reported data for KRT and ICUAW |
| Noh et al., 2013; | No reported data for KRT and ICUAW |
| Nohomovich, Tito, Terrio, & Belardo, 2023; | No reported data for KRT and ICUAW |
| Noi Sedu et al., 2021; | No reported data for KRT and ICUAW |
| Nonoyama, Shigemi, Yasutake, Matsumine, & Ishizuka, 2022; | No reported data for KRT and ICUAW |
| Noutsos, Currie, Lek, & Isbister, 2020; | No reported data for KRT and ICUAW |
| Núñez-Seisdedos, Lázaro-Navas, López-González, & López-Aguilera, 2022; | No reported data for KRT and ICUAW |
| Obata et al., 2017; | No reported data for KRT and ICUAW |
| Oberhuber et al., 2019; | No reported data for KRT and ICUAW |
| Oda et al., 1997; | No reported data for KRT and ICUAW |
| Oishi et al., 2024; | No reported data for KRT and ICUAW |
| Okubo et al., 2023; | No reported data for KRT and ICUAW |
| Oliveros et al., 2020; | No reported data for KRT and ICUAW |
| Olivo Freites, Sy, Miguez, & Salonia, 2022; | No reported data for KRT and ICUAW |
| Ong et al., 2020; | No reported data for KRT and ICUAW |
| Orlitová et al., 2023; | No reported data for KRT and ICUAW |
| Orquera, Pernasetti, Ojeda, Agüero, & Godoy, 2022; | No reported data for KRT and ICUAW |
| Ortega & Solís, 2006; | No reported data for KRT and ICUAW |
| Osgood et al., 2015; | No reported data for KRT and ICUAW |
| Osuna-Padilla, Rodríguez-Moguel, Aguilar-Vargas, & Rodríguez-Llamazares, 2021; | No reported data for KRT and ICUAW |
| Othman, Elbiaa, Mansour, El-Menshawy, & Elsayed, 2023; | No reported data for KRT and ICUAW |
| Oto et al., 2022; | No reported data for KRT and ICUAW |
| Ou et al., 2021; | No reported data for KRT and ICUAW |
| Overmiller & Bitter, 2021; | No reported data for KRT and ICUAW |
| Owais et al., 2023; | No reported data for KRT and ICUAW |
| Ozdemir, Bomkamp, Hyatt, Smuder, & Powers, 2022; | No reported data for KRT and ICUAW |
| Özkale & Özkale, 2022; | No reported data for KRT and ICUAW |
| Pabst, El-Banayosy, Soleimani, & Brehm, 2018; | No reported data for KRT and ICUAW |
| Pakhchanian et al., 2022; | No reported data for KRT and ICUAW |
| Panagiotidou et al., 2018; | No reported data for KRT and ICUAW |
| Panahi, Malekmohammad, Soleymani, & Hashemian, 2020; | No reported data for KRT and ICUAW |
| Panchal & Casadonte, 2020; | No reported data for KRT and ICUAW |
| Pang, Chanouzas, & Baharani, 2014; | No reported data for KRT and ICUAW |
| Paolo et al., 2022; | No reported data for KRT and ICUAW |
| Papadakis, Sapkas, & Tzoutzopoulos, 2008; | No reported data for KRT and ICUAW |
| Papadimitriou et al., 2021; | No reported data for KRT and ICUAW |
| Parauda et al., 2020; | No reported data for KRT and ICUAW |
| Parchem, Peck, & Tales, 2018; | No reported data for KRT and ICUAW |
| J. Park et al., 2015; | No reported data for KRT and ICUAW |
| Y. J. Park & Kim, 2013; | No reported data for KRT and ICUAW |
| Parperis & Al-Ani, 2016; | No reported data for KRT and ICUAW |
| Pasyuga et al., 2020; | No reported data for KRT and ICUAW |
| Patejdl et al., 2019; | No reported data for KRT and ICUAW |
| B. K. Patel et al., 2023; | No reported data for KRT and ICUAW |
| C. Patel, Thompson, Copley-Harris, & Hattab, 2019; | No reported data for KRT and ICUAW |
| M. P. Patel et al., 2015; | No reported data for KRT and ICUAW |
| M. S. Patel et al., 2015; | No reported data for KRT and ICUAW |
| R. Patel, Patel, Mulvoy, & Kapoor, 2017; | No reported data for KRT and ICUAW |
| S. Patel, Bear, Patel, & Puthucheary, 2020; | No reported data for KRT and ICUAW |
| Patle Vidhi, Crawford, Grace, & Edwards, 2012; | No reported data for KRT and ICUAW |
| Patsaki et al., 2017; | No reported data for KRT and ICUAW |
| Patten, Pearn, DeBuse, Burke, & Covacevich, 1985; | No reported data for KRT and ICUAW |
| Patti, Pfefferle, & Myer, 2014; | No reported data for KRT and ICUAW |
| Paul, Sood, Paul, & Puri, 2009; | No reported data for KRT and ICUAW |
| Pavlov, Babić, Bulj, & Krčmar, 2020; | No reported data for KRT and ICUAW |
| Pawson, Jayaweera, & Wigmore, 2008; | No reported data for KRT and ICUAW |
| Pelekhaty & Menaker, 2018; | No reported data for KRT and ICUAW |
| Peñuelas et al., 2018; | No reported data for KRT and ICUAW |
| Péré et al., 2020; | No reported data for KRT and ICUAW |
| Pereira et al., 2023; | No reported data for KRT and ICUAW |
| Perez-Vela et al., 1996; | No reported data for KRT and ICUAW |
| Pérez et al., 2021; | No reported data for KRT and ICUAW |
| Perrin et al., 2021; | No reported data for KRT and ICUAW |
| Pessoa et al., 2019; | No reported data for KRT and ICUAW |
| Petersson, Hansen, Svenningsson, & Lundstrom, 2022; | No reported data for KRT and ICUAW |
| Petri, Hayes, & Schwartzstein, 2020; | No reported data for KRT and ICUAW |
| Peyko, Shams, & Lauver, 2021; | No reported data for KRT and ICUAW |
| Pezzi et al., 2019; | No reported data for KRT and ICUAW |
| Phillips, Dickerson, Moore, Paddon-Jones, & Weijs, 2017; | No reported data for KRT and ICUAW |
| Piatkowski, Gröger, Bozkurt, Fuchs, & Pallua, 2007; | No reported data for KRT and ICUAW |
| Pierre, Thieffry, Moreau, & Duburcq, 2018; | No reported data for KRT and ICUAW |
| Pillarisetti & Ahmed, 2007; | No reported data for KRT and ICUAW |
| Pizzamiglio et al., 2022; | No reported data for KRT and ICUAW |
| Plaut & Weiss, 2024a, 2024b; | No reported data for KRT and ICUAW |
| Polanía-Sandoval et al., 2022; | No reported data for KRT and ICUAW |
| Polastri, Oldani, Pisani, & Nava, 2018; | No reported data for KRT and ICUAW |
| Ponikowski et al., 2016; | No reported data for KRT and ICUAW |
| Ponikvar, 2003; | No reported data for KRT and ICUAW |
| Pothineni, Hayes, Deshmukh, & Paydak, 2015; | No reported data for KRT and ICUAW |
| Poyant et al., 2021; | No reported data for KRT and ICUAW |
| Prasad, Bitla, Manohar, Devi, & Srinivasa Rao, 2012; | No reported data for KRT and ICUAW |
| Pratt, Connell, Bekhit, & Crawford, 2018; | No reported data for KRT and ICUAW |
| Praveen Kumar, Praveen Kumar, & Sharvanan, 2013; | No reported data for KRT and ICUAW |
| Preiser, 2018; | No reported data for KRT and ICUAW |
| Prielipp & Coursin, 2015; | No reported data for KRT and ICUAW |
| Pullara et al., 2013; | No reported data for KRT and ICUAW |
| Puri, Magoon, & Bandyopadhyay, 2023; | No reported data for KRT and ICUAW |
| Puthucheary & Rooyackers, 2022; | No reported data for KRT and ICUAW |
| W. Qi, Murphy, Doyle, & Ferrante, 2023; | No reported data for KRT and ICUAW |
| X. Qi et al., 2020; | No reported data for KRT and ICUAW |
| Qie, Liu, & Guo, 2022 | No reported data for KRT and ICUAW |
| Qiu, Jiang, & Xi, 2019; | No reported data for KRT and ICUAW |
| Queiroz et al., 2020; | No reported data for KRT and ICUAW |
| Quidley, Bookstaver, Gainey, & Gainey, 2012; | No reported data for KRT and ICUAW |
| Quintana-Ortega et al., 2021; | No reported data for KRT and ICUAW |
| Quiroz Alfaro, Rodríguez Acosta, Tanaka Takegami, Bracho Maya, & Quiroz Simanca, 2022; | No reported data for KRT and ICUAW |
| Raasveld et al., 2021; | No reported data for KRT and ICUAW |
| H. Radhakrishnan, 2014; | No reported data for KRT and ICUAW |
| J. Radhakrishnan, Markowitz, & Cohen, 2006; | No reported data for KRT and ICUAW |
| Rahiminezhad, Zakeri, & Dehghan, 2023; | No reported data for KRT and ICUAW |
| Raju et al., 2017; | No reported data for KRT and ICUAW |
| Rakkolainen, Mustonen, & Vuola, 2020; | No reported data for KRT and ICUAW |
| Rakocevic, Alaslani, & Torres, 2022; | No reported data for KRT and ICUAW |
| Ramly, MacFie, Eshraghi, Cole, & Engel, 2018; | No reported data for KRT and ICUAW |
| Ramos-Rossy, Flores, Otero-Domínguez, Torres-Palacios, & Rodríguez-Cintrón, 2018; | No reported data for KRT and ICUAW |
| Ramsi & Al Ali, 2018; | No reported data for KRT and ICUAW |
| Ranjit, Ramanathan, Ramakrishnan, & Kissoon, 2018; | No reported data for KRT and ICUAW |
| Rasche et al., 2006; | No reported data for KRT and ICUAW |
| Rathnayaka & Ranathunga, 2017; | No reported data for KRT and ICUAW |
| Rathnayaka, Ranathunga, & Kularatne, 2021; | No reported data for KRT and ICUAW |
| Rauch et al., 2022; | No reported data for KRT and ICUAW |
| Raurell-Torredà et al., 2021, 2022; | No reported data for KRT and ICUAW |
| Raurell-Torredà et al., 2019; | No reported data for KRT and ICUAW |
| Raymond, Wiesen, Rehm, & Auron, 2014; | No reported data for KRT and ICUAW |
| Razinger, Kozelj, Gorjup, Grenc, & Brvar, 2021; | No reported data for KRT and ICUAW |
| Rebel et al., 2019; | No reported data for KRT and ICUAW |
| Regmi et al., 2023; | No reported data for KRT and ICUAW |
| Rego Silva et al., 2018; | No reported data for KRT and ICUAW |
| Rehan et al., 2023; | No reported data for KRT and ICUAW |
| Rehmann, Enax-Krumova, Meyer-Frießem, & Schlaffke, 2023; | No reported data for KRT and ICUAW |
| Relvas-Silva et al., 2020; | No reported data for KRT and ICUAW |
| Reyes et al., 2022; | No reported data for KRT and ICUAW |
| H. Rhee et al., 2018; | No reported data for KRT and ICUAW |
| H. Rhee et al., 2017; | No reported data for KRT and ICUAW |
| S. Y. Rhee & Kim, 2021; | No reported data for KRT and ICUAW |
| Rhidian, 2011; | No reported data for KRT and ICUAW |
| Ricci & Ronco, 2009; | No reported data for KRT and ICUAW |
| Rieke et al., 2024; | No reported data for KRT and ICUAW |
| Rimer, Chen, Bar-Nathan, & Segev, 2022; | No reported data for KRT and ICUAW |
| Rizvi, Danic, Silver, & LaBond, 2021; | No reported data for KRT and ICUAW |
| Robledo Cadavid, Salgado Flórez, Garcés Echeverri, Ruiz Santacruz, & Hernandez Ortiz, 2023; | No reported data for KRT and ICUAW |
| Rochefort et al., 2021; | No reported data for KRT and ICUAW |
| Rodgers & Kumar, 2016; | No reported data for KRT and ICUAW |
| Rodriguez-Nava et al., 2021; | No reported data for KRT and ICUAW |
| Rodriguez et al., 2022; | No reported data for KRT and ICUAW |
| Roedl et al., 2021; | No reported data for KRT and ICUAW |
| Roest et al., 2023; | No reported data for KRT and ICUAW |
| Rogliano et al., 2020; | No reported data for KRT and ICUAW |
| Rollinson, Connolly, Berlowitz, & Berney, 2022; | No reported data for KRT and ICUAW |
| Rolls et al., 2014; | No reported data for KRT and ICUAW |
| Romero-Dapueto et al., 2019; | No reported data for KRT and ICUAW |
| Romlin et al., 2015; | No reported data for KRT and ICUAW |
| Ronco, 2005; | No reported data for KRT and ICUAW |
| Ronco, Bagshaw, Gibney, & Bellomo, 2008; | No reported data for KRT and ICUAW |
| Roques, Lytrivi, Rusu, Devriendt, & De Bels, 2011; | No reported data for KRT and ICUAW |
| Rosato et al., 2020; | No reported data for KRT and ICUAW |
| Rosen, Robbins-Juarez, & Stevens, 2021; | No reported data for KRT and ICUAW |
| Rosenberg et al., 2019; | No reported data for KRT and ICUAW |
| Rottmann et al., 2023; | No reported data for KRT and ICUAW |
| Rousseau et al., 2021; | No reported data for KRT and ICUAW |
| D. Roy, song, Awad, & Zamudio, 2021; | No reported data for KRT and ICUAW |
| N. Roy, 2015; | No reported data for KRT and ICUAW |
| Rozemeijer et al., 2021; | No reported data for KRT and ICUAW |
| Rudis et al., 1997 | Includes therapeutic electrical muscle stimulation and no reported data on KRT |
| Rudra, Lin, Miller, Du, & Zhang, 2022; | No reported data for KRT and ICUAW |
| Ruiz, Rosciani, Bisso, & Heras, 2022; | No reported data for KRT and ICUAW |
| Ruwanpathirana & Priyankara, 2022; | No reported data for KRT and ICUAW |
| Saccheri et al., 2020; | No reported data for KRT and ICUAW |
| Safan et al., 2023; | No reported data for KRT and ICUAW |
| Şahintürk, Yurtsever, Ersoy, Kibaroğlu, & Zeyneloğlu, 2021; | No reported data for KRT and ICUAW |
| Sahni, Garg, Garg, Agarwal, & Singh, 2008; | No reported data for KRT and ICUAW |
| Said, Gouvoussis, & Tong, 2023; | No reported data for KRT and ICUAW |
| Saif & Pick, 2021; | No reported data for KRT and ICUAW |
| Saini et al., 2022; | No reported data for KRT and ICUAW |
| Saitoh et al., 2021; | No reported data for KRT and ICUAW |
| Sakai, Hoshino, Nakano, Fujiwara, & Okawa, 2022; | No reported data for KRT and ICUAW |
| Sakan et al., 2013; | No reported data for KRT and ICUAW |
| Sakurai et al., 2023; | No reported data for KRT and ICUAW |
| Sakuraoka, da Silva Boteon, Brown, & Perera, 2019; | No reported data for KRT and ICUAW |
| Salam et al., 2017; | No reported data for KRT and ICUAW |
| Saluja, Swami, Chittora, & Vimlani, 2019; | No reported data for KRT and ICUAW |
| Sampley, Sakhuja, Bhasin, Singh, & Singh, 2020; | No reported data for KRT and ICUAW |
| Samuel, Bajwa, & Cury, 2011; | No reported data for KRT and ICUAW |
| Sánchez et al., 2012; | No reported data for KRT and ICUAW |
| Sangani et al., 2021; | No reported data for KRT and ICUAW |
| Sanghavi, Aneman, Parr, Dunlops, & Champion, 2006; | No reported data for KRT and ICUAW |
| Saour et al., 2014; | No reported data for KRT and ICUAW |
| Sapra, 2021; | No reported data for KRT and ICUAW |
| Sarfati et al., 2018; | No reported data for KRT and ICUAW |
| Sarıkaya, Direk, Özsoylu, Dursun, & Hatipoğlu, 2022; | No reported data for KRT and ICUAW |
| Sasano, Yasuda, & Yamada, 2022; | No reported data for KRT and ICUAW |
| Sathiavageesan et al., 2022; | No reported data for KRT and ICUAW |
| Saunders, Al Khalifa, Espinosa, & Jain, 2021; | No reported data for KRT and ICUAW |
| Savić, Pejović, & Savić, 2022; | No reported data for KRT and ICUAW |
| Sawalha & Kakkera, 2020; | No reported data for KRT and ICUAW |
| Saxena, Singh, & Verma, 2014; | No reported data for KRT and ICUAW |
| Saydam & Serefli, 2021; | No reported data for KRT and ICUAW |
| Scharf et al., 2021; | No reported data for KRT and ICUAW |
| Scharman & Troutman, 2013; | No reported data for KRT and ICUAW |
| Schefold et al., 2019; | No reported data for KRT and ICUAW |
| Schellenberg et al., 2023; | No reported data for KRT and ICUAW |
| D. Schmidt et al., 2023; | Data included in study Schmidt 2022 |
| M. Schmidt et al., 2019; | No reported data for KRT and ICUAW |
| Scholle, Zierz, Mawrin, Wickenhauser, & Urban, 2020; | No reported data for KRT and ICUAW |
| Schreiber et al., 2014; | No reported data for KRT and ICUAW |
| Scullen, Keen, Mathkour, Dumont, & Kahn, 2020; | No reported data for KRT and ICUAW |
| Secombe & Milne, 2016; | No reported data for KRT and ICUAW |
| Secreto et al., 2023; | No reported data for KRT and ICUAW |
| Segaran, Wandrag, Stotz, Terblanche, & Hickson, 2017; | No reported data for KRT and ICUAW |
| Segers et al., 2014; | Study of therapeutic intervention to minimize neuromuscular dysfunction |
| Selewski & Symons, 2014; | No reported data for KRT and ICUAW |
| Sellorsm, Jones, & Chan, 2014; | No reported data for KRT and ICUAW |
| Serbest, Belhan, Gürger, & Tosun, 2015; | No reported data for KRT and ICUAW |
| Sethi & Vithayathil, 2017; | No reported data for KRT and ICUAW |
| Sha, Qi, & Qindong, 2024; | No reported data for KRT and ICUAW |
| Shabana, Anis, & Ibrahim, 2021; | No reported data for KRT and ICUAW |
| Shadnia, Moiensadat, & Abdollahi, 2004; | No reported data for KRT and ICUAW |
| Shafie, Shahmohamadi, Ghasemi, Zarei Jalalabadi, & Parsa, 2023; | No reported data for KRT and ICUAW |
| H. V. Shah, Irvine, & Bradley, 2008; | No reported data for KRT and ICUAW |
| N. N. Shah & Wilson, 2016; | No reported data for KRT and ICUAW |
| N. R. Shah, Sharma, Waters, Arango, & Scuderi, 2015; | No reported data for KRT and ICUAW |
| P. Shah et al., 2022; | No reported data for KRT and ICUAW |
| S. O. Shah et al., 2018; | No reported data for KRT and ICUAW |
| T. Shah et al., 2023; | No reported data for KRT and ICUAW |
| Shahani & Khardori, 2014; | No reported data for KRT and ICUAW |
| Shan, Dalal, Nahass, Rodricks, & Teichman, 2020; | No reported data for KRT and ICUAW |
| Shankar & Alam, 2022; | No reported data for KRT and ICUAW |
| Shanmugam, Thenappan, & Narasimhan, 2010; | No reported data for KRT and ICUAW |
| Shao et al., 2020; | No reported data for KRT and ICUAW |
| Sharif et al., 2024; | No reported data for KRT and ICUAW |
| Sharma et al., 2016; | No reported data for KRT and ICUAW |
| Shastri, Gupta, & Kumar, 2020; | No reported data for KRT and ICUAW |
| Shetty et al., 2021; | No reported data for KRT and ICUAW |
| Shi et al., 2023; | No reported data for KRT and ICUAW |
| Shimaoka, Atagi, Takaki, & Satani, 1996; | No reported data for KRT and ICUAW |
| Shin, Lim, & Roh, 2020; | No reported data for KRT and ICUAW |
| Shinohara et al., 2023; | No reported data for KRT and ICUAW |
| Shirado et al., 2021; | No reported data for KRT and ICUAW |
| Shokoohi, Smith, Holmes, & Abell, 2008; | No reported data for KRT and ICUAW |
| Shou et al., 2021; | No reported data for KRT and ICUAW |
| Shuai et al., 2018; | No reported data for KRT and ICUAW |
| Sia, Huang, Ueng, Wu, & Chan, 2008; | No reported data for KRT and ICUAW |
| Siddiqui et al., 2022; | No reported data for KRT and ICUAW |
| Sidiras et al., 2019; | No reported data for KRT and ICUAW |
| Şık & Çıtak, 2016; | No reported data for KRT and ICUAW |
| Sillix & McDonald, 1987; | No reported data for KRT and ICUAW |
| Silva et al., 2017; | No reported data for KRT and ICUAW |
| Silva et al., 2018; | No reported data for KRT and ICUAW |
| Simsek & Koca, 2022; | No reported data for KRT and ICUAW |
| Singh et al., 2021; | No reported data for KRT and ICUAW |
| Sion-Sarid et al., 2010; | No reported data for KRT and ICUAW |
| Skinner, Hardcastle, Rodseth, & Muckart, 2014; | No reported data for KRT and ICUAW |
| Smailes, Eagan, Matanle, & Barnes, 2021; | No reported data for KRT and ICUAW |
| Small et al., 2017; | No reported data for KRT and ICUAW |
| Smith & Goslin, 2013; | No reported data for KRT and ICUAW |
| Smithers et al., 2023; | No reported data for KRT and ICUAW |
| Snelson et al., 2017; | No reported data for KRT and ICUAW |
| Sokolov, Hadavi, Mantoan Ritter, & Brunnhuber, 2020; | No reported data for KRT and ICUAW |
| H. W. Song, Shin, Ko, Gwak, & Kim, 2012; | No reported data for KRT and ICUAW |
| Y. H. Song, Seo, Yoo, & Jo, 2019; | No reported data for KRT and ICUAW |
| Soni et al., 2023; | No reported data for KRT and ICUAW |
| Spinale, Laskin, Sondheimer, Swartz, & Goldstein, 2013; | No reported data for KRT and ICUAW |
| Splendiani et al., 2001; | No reported data for KRT and ICUAW |
| Sridharan et al., 2020; | No reported data for KRT and ICUAW |
| Srinivas, Agarwal, & Gupta, 2007; | No reported data for KRT and ICUAW |
| Stephens, Thomas, Ward, & Currie, 2016; | No reported data for KRT and ICUAW |
| Stevens & Zink, 2017; | No reported data for KRT and ICUAW |
| Stewart et al., 2013; | No reported data for KRT and ICUAW |
| Stolldorf, Dietrich, Chidume, McIntosh, & Maxwell, 2018; | No reported data for KRT and ICUAW |
| Stolwijk et al., 2022; | No reported data for KRT and ICUAW |
| Storm et al., 2009; | No reported data for KRT and ICUAW |
| Strachan & Morris, 2017; | No reported data for KRT and ICUAW |
| Studemeister, Studemeister, & Brun, 2018; | No reported data for KRT and ICUAW |
| Subedi, Chowdhury, Tanovic, & Dumic, 2019; | No reported data for KRT and ICUAW |
| Sulaiman, Ismail, Jalalonmuhali, Atiya, & Ponnampalavanar, 2014; | No reported data for KRT and ICUAW |
| Sullivan & Woo, 2018; | No reported data for KRT and ICUAW |
| B. Sun & Li, 2023; | No reported data for KRT and ICUAW |
| K. Sun et al., 2023; | No reported data for KRT and ICUAW |
| L. Sun, Zhou, Tian, Zhang, & Guo, 2021; | No reported data for KRT and ICUAW |
| L. Z. Sun et al., 2009; | No reported data for KRT and ICUAW |
| R. Sun, Fang, Jiang, Yu, & Tao, 2023; | No reported data for KRT and ICUAW |
| W. Sun et al., 2021; | No reported data for KRT and ICUAW |
| Supinski et al., 2021; | No reported data for KRT and ICUAW |
| Surabotsophon et al., 2020; | No reported data for KRT and ICUAW |
| Sy-Go et al., 2021; | No reported data for KRT and ICUAW |
| Szajek et al., 2021; | No reported data for KRT and ICUAW |
| Szawarski, 2016; | No reported data for KRT and ICUAW |
| Szczanowicz et al., 2021; | No reported data for KRT and ICUAW |
| Tagliaferri, Pjetergjoka, & Leou, 2022; | No reported data for KRT and ICUAW |
| Taketa, Uchiyama, Kodama, Koyama, & Domen, 2023; | No reported data for KRT and ICUAW |
| Takino et al., 2023; | No reported data for KRT and ICUAW |
| Taleb Abdellah & Koratala, 2022; | No reported data for KRT and ICUAW |
| Tang et al., 2013; | No reported data for KRT and ICUAW |
| Taniguchi et al., 2021; | No reported data for KRT and ICUAW |
| Tankisi et al., 2021; | No reported data for KRT and ICUAW |
| Tantibhedhyangkul et al., 2020; | No reported data for KRT and ICUAW |
| Tatsumi et al., 2021; | No reported data for KRT and ICUAW |
| G. S. Tavares, Oliveira, Mendes, & Velloso, 2023; | No reported data for KRT and ICUAW |
| L. C. B. Tavares et al., 2020; | No reported data for KRT and ICUAW |
| Taxbro, Kahlow, Wulcan, & Fornarve, 2020; | No reported data for KRT and ICUAW |
| Teixeira et al., 2023; | No reported data for KRT and ICUAW |
| Tekin, Özdoğan, Demir, Soultan, & Zafar, 2021; | No reported data for KRT and ICUAW |
| Tepper et al., 2000; | Case series |
| Terano et al., 2018; | No reported data for KRT and ICUAW |
| Tessitore et al., 2021; | No reported data for KRT and ICUAW |
| Thackeray, Mohebbi, Orford, Kotowicz, & Pasco, 2021; | No reported data for KRT and ICUAW |
| Thadchanamoorthy & Dayasiri, 2022; | No reported data for KRT and ICUAW |
| Thery, Cousin, Tissieres, Enault, & Morin, 2022; | No reported data for KRT and ICUAW |
| Thiele et al., 1997; | No reported data for KRT and ICUAW |
| Thiele et al., 2000; | No reported data for KRT and ICUAW |
| Thille et al., 2020; | No reported data for KRT and ICUAW |
| K. Thomas, Majuran, & Thomas, 2018; | No reported data for KRT and ICUAW |
| S. Thomas & Mehrholz, 2018; | No reported data for KRT and ICUAW |
| S. Thomas, Mehrholz, Bodechtel, & Elsner, 2019; | No reported data for KRT and ICUAW |
| S. Thomas, Sauter, Starrost, Pohl, & Mehrholz, 2017, 2018; | No reported data for KRT and ICUAW |
| Togashi et al., 2020; | No reported data for KRT and ICUAW |
| Tokuhira et al., 2012; | No reported data for KRT and ICUAW |
| Torre et al., 2023; | No reported data for KRT and ICUAW |
| Torres et al., 2017; | No reported data for KRT and ICUAW |
| Tracy, Lynch, Messenger, Vaden, & Vigani, 2022; | No reported data for KRT and ICUAW |
| A. Tran, Walsh, Batt, Dos Santos, & Hu, 2020; | No reported data for KRT and ICUAW |
| D. H. Tran et al., 2023; | No reported data for KRT and ICUAW |
| Traube, Augenstein, Greenwald, LaQuaglia, & Silver, 2014; | No reported data for KRT and ICUAW |
| Traugott et al., 2021; | No reported data for KRT and ICUAW |
| Trieu et al., 2021; | No reported data for KRT and ICUAW |
| Trifi et al., 2016; | No reported data for KRT and ICUAW |
| Trujillo, Bellorin-Font, Fragachan, & Perret-Gentil, 2009; | No reported data for KRT and ICUAW |
| Tsai et al., 2013; | No reported data for KRT and ICUAW |
| Tseng et al., 2016a, 2016b; | No reported data for KRT and ICUAW |
| Tsukinoki & Murakami, 2013; | No reported data for KRT and ICUAW |
| Tsutsumi et al., 2021; | No reported data for KRT and ICUAW |
| Tuero et al., 2018; | No reported data for KRT and ICUAW |
| Tufan-Pekkucuksen et al., 2018; | No reported data for KRT and ICUAW |
| Tumian & Wong, 2015; | No reported data for KRT and ICUAW |
| Ulutaş, S, & Ardıç, 2021; | No reported data for KRT and ICUAW |
| Umapathi et al., 2020; | No reported data for KRT and ICUAW |
| Umman et al., 2022; | No reported data for KRT and ICUAW |
| Uncini et al., 2021; | No reported data for KRT and ICUAW |
| Usuda et al., 2023; | No reported data for KRT and ICUAW |
| Uz et al., 2022; | No reported data for KRT and ICUAW |
| Vaidya & Acevedo, 2015; | No reported data for KRT and ICUAW |
| Valero et al., 2022; | No reported data for KRT and ICUAW |
| Valsalan et al., 2017; | No reported data for KRT and ICUAW |
| Van Aerde, Meersseman, et al., 2020; | No reported data for KRT and ICUAW |
| Van Aerde, Van den Berghe, Wilmer, Gosselink, & Hermans, 2020; | No reported data for KRT and ICUAW |
| Van De Wyngaert, Dewulf, Collienne, Laterre, & Hantson, 2022; | No reported data for KRT and ICUAW |
| Van Den Berghe et al., 2005; | Data included in study Weber-Carstens 2009 |
| Van den Berghe et al., 2003; | Data included in study Weber-Carstens 2009 |
| Van Der Meer, Conway, Little, & Hanson, 2020; | No reported data for KRT and ICUAW |
| van Oorsouw et al., 2022; | No reported data for KRT and ICUAW |
| van Wagenberg, Witteveen, Wieske, & Horn, 2020; | No reported data for KRT and ICUAW |
| Vanhorebeek et al., 2023; | No reported data for KRT and ICUAW |
| Vankrunkelsven et al., 2022; | No reported data for KRT and ICUAW |
| Vattoth, Abdelhady, Alsoub, Own, & Elsotouhy, 2020; | No reported data for KRT and ICUAW |
| Vazin, Mahi Birjand, & Darake, 2018; | No reported data for KRT and ICUAW |
| Veldema et al., 2019; | No reported data for KRT and ICUAW |
| Venkategowda, Prakash, Harde, & Rao, 2015; | No reported data for KRT and ICUAW |
| Venugopal, Mallikarjun Reddy, Bharathraj, Jaligidad, & Kushal, 2014; | No reported data for KRT and ICUAW |
| Vercek et al., 2019; | No reported data for KRT and ICUAW |
| Verceles et al., 2023; | No reported data for KRT and ICUAW |
| Verstraeten, Verbrugghe, & Wuyts, 2013; | No reported data for KRT and ICUAW |
| Vigneri et al., 2014; | No reported data for KRT and ICUAW |
| Vijayan et al., 2021; | No reported data for KRT and ICUAW |
| Vikrant, Jaryal, Gupta, & Parashar, 2019; | No reported data for KRT and ICUAW |
| Viktorsdottir, Indridason, & Palsson, 2014; | No reported data for KRT and ICUAW |
| Vinod, Srikant, Thiruvikramaprakash, & Dutta, 2015; | No reported data for KRT and ICUAW |
| Vordoni, Theofilis, Vlachopanos, Koukoulaki, & Kalaitzidis, 2021; | No reported data for KRT and ICUAW |
| Voros, Osvath, Fekete, & Tenyi, 2008; | No reported data for KRT and ICUAW |
| Vreeswijk, Arbous, Matta, & van Westerloo, 2020; | No reported data for KRT and ICUAW |
| Vrsalovic, Tesovic, & Mise, 2007; | No reported data for KRT and ICUAW |
| Vuillard et al., 2018; | No reported data for KRT and ICUAW |
| Vukovic, Meier, Guligowska, & Zalizko, 2023; | No reported data for KRT and ICUAW |
| Wada et al., 2020; | No reported data for KRT and ICUAW |
| Wainstein et al., 2022; | No reported data for KRT and ICUAW |
| Wainstein et al., 2023; | No reported data for KRT and ICUAW |
| Wakabayashi, Kikuno, Ohwada, & Kikawada, 1994; | No reported data for KRT and ICUAW |
| Wakuda et al., 2021; | No reported data for KRT and ICUAW |
| Waldauf et al., 2019; | No reported data for KRT and ICUAW |
| Walsh, Batt, et al., 2022; | No reported data for KRT and ICUAW |
| Walsh, Escudero King, et al., 2022; | No reported data for KRT and ICUAW |
| Wan Azman, Sukor, Abu Shamsi, Ismail, & Kamaruddin, 2022; | No reported data for KRT and ICUAW |
| Wandrag et al., 2019; | No reported data for KRT and ICUAW |
| B. Wang et al., 2022; | No reported data for KRT and ICUAW |
| J. Wang & Wu, 2020; | No reported data for KRT and ICUAW |
| J. L. Wang et al., 2020; | No reported data for KRT and ICUAW |
| L. Wang, Lyu, Shen, Jin, & Sheng, 2021; | No reported data for KRT and ICUAW |
| L. Wang, Zhang, Wu, & Zhou, 2023; | No reported data for KRT and ICUAW |
| M. Wang et al., 2021; | No reported data for KRT and ICUAW |
| Q. Wang, Fu, Su, & Fu, 2022; | No reported data for KRT and ICUAW |
| Q. P. Wang et al., 2022; | No reported data for KRT and ICUAW |
| S. L. Wang et al., 2020; | No reported data for KRT and ICUAW |
| X. Wang et al., 2022; | No reported data for KRT and ICUAW |
| X. Wang et al., 2020; | No reported data for KRT and ICUAW |
| Y. Wang, Sun, Ren, & Guo, 2021; | No reported data for KRT and ICUAW |
| Y. T. Wang et al., 2023; | No reported data for KRT and ICUAW |
| Z. Wang et al., 2020; | No reported data for KRT and ICUAW |
| Watanabe et al., 2018; | No reported data for KRT and ICUAW |
| S. Watanabe et al., 2019; | No reported data for KRT and ICUAW |
| Y. Watanabe et al., 2019; | No reported data for KRT and ICUAW |
| Wcs, Jpk, Yw, & Yc, 2016; | No reported data for KRT and ICUAW |
| Wei, Jiang, Wang, He, & Li, 2018; | No reported data for KRT and ICUAW |
| T. Wen et al., 2023; | No reported data for KRT and ICUAW |
| Y. K. Wen, 2009; | No reported data for KRT and ICUAW |
| Wernhart et al., 2021; | No reported data for KRT and ICUAW |
| Wibart et al., 2023; | No reported data for KRT and ICUAW |
| M. E. Wilson et al., 2018; | No reported data for KRT and ICUAW |
| W. Wilson, Ali-Osman, Sucher, Shirah, & Mangram, 2019; | No reported data for KRT and ICUAW |
| Wiltshire & Custer, 2003; | No reported data for KRT and ICUAW |
| Wimmer et al., 2022; | No reported data for KRT and ICUAW |
| Windpessl et al., 2014; | No reported data for KRT and ICUAW |
| Witteveen, Sommers, et al., 2017; | No reported data for KRT and ICUAW |
| Witteveen et al., 2018; | No reported data for KRT and ICUAW |
| Witteveen et al., 2019; | No reported data for KRT and ICUAW |
| Witteveen et al., 2020; | No reported data for KRT and ICUAW |
| Witteveen, Wieske, et al., 2017; | No reported data for KRT and ICUAW |
| E. M. Wolfe et al., 2022; | No reported data for KRT and ICUAW |
| K. S. Wolfe et al., 2018; | No reported data for KRT and ICUAW |
| Wollersheim et al., 2019; | No reported data for KRT and ICUAW |
| Wollersheim et al., 2017; | No reported data for KRT and ICUAW |
| C. Wong, Hsu, & Carr, 2015; | No reported data for KRT and ICUAW |
| C. K. Wong, Hamizah, Baherin, & Lee, 2020; | No reported data for KRT and ICUAW |
| C. S. Wu, Tong, Ong, & Sung, 2015; | No reported data for KRT and ICUAW |
| G. Wu & Zhou, 2021; | No reported data for KRT and ICUAW |
| H. H. L. Wu, Chan, Lau, & Yan, 2010; | No reported data for KRT and ICUAW |
| T. T. Wu et al., 2023; | No reported data for KRT and ICUAW |
| Y. Wu et al., 2020; | No reported data for KRT and ICUAW |
| Y. Wu et al., 2021; | No reported data for KRT and ICUAW |
| Xiao, Ran, Zhong, Le, & Li, 2022; | No reported data for KRT and ICUAW |
| Xie, Bowe, Maddukuri, & Al-Aly, 2020; | No reported data for KRT and ICUAW |
| Xie, Liu, et al., 2020; | No reported data for KRT and ICUAW |
| J. Xing et al., 2020; | No reported data for KRT and ICUAW |
| L. Xing et al., 2021; | No reported data for KRT and ICUAW |
| J. Xu et al., 2022; | No reported data for KRT and ICUAW |
| Y. Xu et al., 2021; | No reported data for KRT and ICUAW |
| Xuri, Qing, Lisui, Tinglong, & Yuqi, 2020; | No reported data for KRT and ICUAW |
| Yaghmaii, Nili, Najafi, Cheloi, & Moghtaderi, 2022; | No reported data for KRT and ICUAW |
| Yaman, 2022; | No reported data for KRT and ICUAW |
| H. T. Yang et al., 2014; | No reported data for KRT and ICUAW |
| R. Yang, Moosavi, Eppanapally, Aboeed, & Munoz, 2020; | No reported data for KRT and ICUAW |
| T. Yang et al., 2018; | No reported data for KRT and ICUAW |
| T. Yang et al., 2020; | No reported data for KRT and ICUAW |
| T. Yang, Li, Jiang, & Xi, 2021; | No reported data for KRT and ICUAW |
| T. Y. Yang et al., 2009; | No reported data for KRT and ICUAW |
| W. Yang & Zhang, 2021; | No reported data for KRT and ICUAW |
| X. Yang et al., 2022; | No reported data for KRT and ICUAW |
| Y. Yang et al., 2021; | No reported data for KRT and ICUAW |
| Yazaki, Sakuma, Hikita, Fujimaru, & Hamazaki, 2022; | No reported data for KRT and ICUAW |
| Yeganeh et al., 2023; | No reported data for KRT and ICUAW |
| Yeoh, Ng, & Goh, 2017; | No reported data for KRT and ICUAW |
| Yeşilbaş, Kıhtır, Yıldırım, Hatipoğlu, & Şevketoğlu, 2016; | No reported data for KRT and ICUAW |
| J. C. Yeung, Cypel, Chaparro, & Keshavjee, 2021; | No reported data for KRT and ICUAW |
| M. T. Yeung et al., 2023; | No reported data for KRT and ICUAW |
| Yoshinaka, Akatsuka, Yamamoto, & Yamakage, 2021; | No reported data for KRT and ICUAW |
| Yoshiya et al., 2021; | No reported data for KRT and ICUAW |
| Young, Tay, Lau, & Huen, 2014; | No reported data for KRT and ICUAW |
| G. Yu et al., 2022; | No reported data for KRT and ICUAW |
| L. Yu, Jiang, Zhang, Chen, & Shi, 2020; | No reported data for KRT and ICUAW |
| X. Yu, Wan, Wan, & Huang, 2018; | No reported data for KRT and ICUAW |
| Y. Yu et al., 2023; | No reported data for KRT and ICUAW |
| Y. Yu et al., 2020; | No reported data for KRT and ICUAW |
| G. Yuan, Zhang, Mou, Luo, & Xie, 2021; | No reported data for KRT and ICUAW |
| H. Yuan, Liu, Gao, & Hu, 2021; | No reported data for KRT and ICUAW |
| S. Y. Yuan, Xie, & Yang, 2021; | No reported data for KRT and ICUAW |
| Z. Yuan, Pan, Wang, & Wang, 2022; | No reported data for KRT and ICUAW |
| Yurtseven, Türksoylu, Yazıcı, Karapınar, & Saz, 2018; | No reported data for KRT and ICUAW |
| Zahid et al., 2020; | No reported data for KRT and ICUAW |
| Zaidan et al., 2012; | No reported data for KRT and ICUAW |
| Zakeri, Aziz, Rahiminezhad, & Dehghan, 2023; | No reported data for KRT and ICUAW |
| Zakynthinos et al., 2001; | No reported data for KRT and ICUAW |
| Zamoner et al., 2021; | No reported data for KRT and ICUAW |
| Zanders et al., 2022; | No reported data for KRT and ICUAW |
| Zaragoza-García et al., 2023; | No reported data for KRT and ICUAW |
| Zayed, Osman, Kheiri, Azher, & Bachuwa, 2019; | No reported data for KRT and ICUAW |
| Zeiad et al., 2021; | No reported data for KRT and ICUAW |
| Zeng et al., 2021; | No reported data for KRT and ICUAW |
| Zengin et al., 2015; | No reported data for KRT and ICUAW |
| A. Zhang et al., 2022; | No reported data for KRT and ICUAW |
| F. Zhang et al., 2022; | No reported data for KRT and ICUAW |
| L. Zhang et al., 2012; | No reported data for KRT and ICUAW |
| W. Zhang et al., 2021; | No reported data for KRT and ICUAW |
| Y. Zhang et al., 2021; | No reported data for KRT and ICUAW |
| Z. Zhang, G. Wang, et al., 2021; | No reported data for KRT and ICUAW |
| Z. Zhang, Yang, & Luo, 2021; | No reported data for KRT and ICUAW |
| K. Zhao et al., 2022; | No reported data for KRT and ICUAW |
| R. Zhao et al., 2020; | No reported data for KRT and ICUAW |
| Z. Zhao, Chu, Chang, Chang, & Hsu, 2018; | No reported data for KRT and ICUAW |
| Zheng et al., 2020; | No reported data for KRT and ICUAW |
| Zhi et al., 2020; | No reported data for KRT and ICUAW |
| H. Zhou et al., 2019; | No reported data for KRT and ICUAW |
| J. Zhou, Zhang, Zhou, & Zhang, 2022; | No reported data for KRT and ICUAW |
| W. Zhou, Shi, Fan, & Zhu, 2020; | No reported data for KRT and ICUAW |
| W. Zhou et al., 2022; | No reported data for KRT and ICUAW |
| X. Zhou, Jin, Liu, & Duan, 2023; | No reported data for KRT and ICUAW |
| C. Zhu et al., 2018; | No reported data for KRT and ICUAW |
| D. C. Zhu et al., 2022; | No reported data for KRT and ICUAW |
| J. M. Zhu et al., 2016; | No reported data for KRT and ICUAW |
| Zinder, Andrews, Cristallo, & Flattau, 2023; | No reported data for KRT and ICUAW |
| Zobel, Ring, Trop, & Trittenwein, 1986; | No reported data for KRT and ICUAW |
| Zoetman, Paramarta, Bouman, Sanou, & Vlaar, 2018; | No reported data for KRT and ICUAW |
| Zorko, Samaan, & Gupta, 2021; | No reported data for KRT and ICUAW |
| Zou, Liu, & Li, 2018; | No reported data for KRT and ICUAW |
| Zubkov, Rabinstein, Manno, & Wijdicks, 2008; | No reported data for KRT and ICUAW |
| Zuercher, Moret, & Schefold, 2019; | No reported data for KRT and ICUAW |
| Župan et al., 2017; | No reported data for KRT and ICUAW |

Abbreviations: ICUAW – intensive care unit acquired weakness; KRT - kidney replacement therapy
