# Supplementary material for: Incidence of intensive care unit acquired weakness in critically ill patients treated with kidney replacement therapy: A systematic review and meta-analysis
Source: PLoS One. 2025 May 15;20(5):e0323874. doi: 10.1371/journal.pone.0323874 (PMC12080829; doi:10.1371/journal.pone.0323874)
Supplement: S3 Table — (DOCX) [file pone.0323874.s003.docx]

**S3-Table- Data extraction of included studies**

| **Author/Year** | **Meets Inclusion Criteria?** | **Data Extractor Name and Date** | **ICUAW (n);**  **No ICUAW (n);**  **Use of KRT/Not** |
| --- | --- | --- | --- |
| Watanabe, S. 2023 | Y | Yang Tao, Zhang Kaikai; August 2024 | 25 vs 37  15 vs 66  40 vs 103 |
| Yamada, K. 2023 | Y | Yang Tao, Zhang Kaikai; August 2024 | 8 vs 72  1 vs 76  9 vs 148 |
| Schmidt, D. 2022 | Y | Yang Tao, Zhang Kaikai; August 2024 | 13 vs 15  6 vs 41  19 vs 56 |
| Frithiof, R. 2021 | Y | Yang Tao, Zhang Kaikai; August 2024 | 7 vs 4  8 vs 92  15 vs 96 |
| Hermans, G. 2009 | Y | Yang Tao, Zhang Kaikai; August 2024 | 118 vs 183  72 vs 168  190 vs 351 |
| Yang, Z. 2023 | Y | Yang Tao, Zhang Kaikai; August 2024 | 9 vs 31  50 vs 190  59 vs 221 |
| Nanas, S. 2008 | Y | Yang Tao, Zhang Kaikai; August 2024 | 16 vs 28  31 vs 110  47 vs 138 |
| Garnacho-Montero, J. 2001 | Y | Yang Tao, Zhang Kaikai; August 2024 | 10 vs 40  11 vs 12  21 vs 52 |
| Campellone, J. V. 1998 | Y | Yang Tao, Zhang Kaikai; August 2024 | 4 vs 3  13 vs 67  17 vs 70 |
| De Jonghe, B. 2002 | Y | Yang Tao, Zhang Kaikai; August 2024 | 8 vs 16  9 vs 62  17 vs 78 |
| Liu, J. 2024 | Y | Yang Tao, Zhang Kaikai; August 2024 | 53 vs 61  57 vs 93  110 vs 154 |
| Chen, L. 2024 | Y | Yang Tao, Zhang Kaikai; August 2024 | 19 vs 97  23 vs 125  42 vs 222 |

Abbreviations: ICUAW – intensive care unit acquired weakness; KRT - kidney replacement therapy
