# Supplementary material for: Incidence of intensive care unit acquired weakness in critically ill patients treated with kidney replacement therapy: A systematic review and meta-analysis
Source: PLoS One. 2025 May 15;20(5):e0323874. doi: 10.1371/journal.pone.0323874 (PMC12080829; doi:10.1371/journal.pone.0323874)
Supplement: S1 File — (DOCX) [file pone.0323874.s004.docx]

**Search Strategy PubMed**

**Domain: ICU patients**

("Critical Care"[MeSH Terms] OR ("critical"[All Fields] AND "care"[All Fields]) OR "Critical Care"[All Fields] OR ("Intensive Care Units"[MeSH Terms] OR ("intensive"[All Fields] AND "care"[All Fields] AND "units"[All Fields]) OR "Intensive Care Units"[All Fields] OR "icu"[All Fields]) OR ("Critical Care"[MeSH Terms] OR ("critical"[All Fields] AND "care"[All Fields]) OR "Critical Care"[All Fields] OR ("intensive"[All Fields] AND "care"[All Fields]) OR "intensive care"[All Fields]) OR (("critical"[All Fields] OR "critically"[All Fields]) AND "ill"[All Fields]) OR ("Critical Illness"[MeSH Terms] OR ("critical"[All Fields] AND "illness"[All Fields]) OR "Critical Illness"[All Fields]) OR ("Critical Illness"[MeSH Terms] OR ("critical"[All Fields] AND "illness"[All Fields]) OR "Critical Illness"[All Fields] OR ("critically"[All Fields] AND "ill"[All Fields]) OR "critically ill"[All Fields]) OR ("Intensive Care Units"[MeSH Terms] OR "Critical Care"[MeSH Terms] OR "Critical Illness"[MeSH Terms]))

**Determinant：**

(RRT OR renal replacement therapy) OR " renal replacement therapy"[MeSH Terms]

**Outcome: Weakness**

("Muscular Diseases"[MeSH Terms] OR "Muscle Weakness"[MeSH Terms] OR "Polyneuropathies"[MeSH Terms] OR "Paralysis"[MeSH Terms] OR "Paresis"[MeSH Terms] OR "Neuromuscular Diseases"[MeSH Terms] OR ("paralysing"[All Fields] OR "Paralysis"[MeSH Terms] OR "Paralysis"[All Fields] OR "paralyse"[All Fields] OR "paralysed"[All Fields] OR "paralyses"[All Fields] OR ("Paresis"[MeSH Terms] OR "Paresis"[All Fields] OR "pareses"[All Fields]) OR ("quadriplegia"[MeSH Terms] OR "quadriplegia"[All Fields] OR "quadriplegias"[All Fields]) OR "weakness"[All Fields] OR "weaknesses"[All Fields]) OR ("Muscular Diseases"[MeSH Terms] OR ("muscular"[All Fields] AND "diseases"[All Fields]) OR "Muscular Diseases"[All Fields] OR ("muscular"[All Fields] AND "disease"[All Fields]) OR "muscular disease"[All Fields]) OR ("Muscular Diseases"[MeSH Terms] OR ("muscular"[All Fields] AND "diseases"[All Fields]) OR "Muscular Diseases"[All Fields]) OR ("Neuromuscular Diseases"[MeSH Terms] OR ("neuromuscular"[All Fields] AND "diseases"[All Fields]) OR "Neuromuscular Diseases"[All Fields] OR ("neuromuscular"[All Fields] AND "disease"[All Fields]) OR "neuromuscular disease"[All Fields]) OR ("Neuromuscular Diseases"[MeSH Terms] OR ("neuromuscular"[All Fields] AND "diseases"[All Fields]) OR "Neuromuscular Diseases"[All Fields]) OR ("Muscular Diseases"[MeSH Terms] OR ("muscular"[All Fields] AND "diseases"[All Fields]) OR "Muscular Diseases"[All Fields] OR "myopathies"[All Fields] OR "myopathy"[All Fields]) OR ("Muscular Diseases"[MeSH Terms] OR ("muscular"[All Fields] AND "diseases"[All Fields]) OR "Muscular Diseases"[All Fields] OR "myopathies"[All Fields] OR "myopathy"[All Fields]) OR ("neuropathies"[All Fields] OR "neuropathy"[All Fields]) OR ("neuropathies"[All Fields] OR "neuropathy"[All Fields]) OR ("Polyneuropathies"[MeSH Terms] OR "Polyneuropathies"[All Fields] OR "polyneuropathy"[All Fields]) OR ("Polyneuropathies"[MeSH Terms] OR "Polyneuropathies"[All Fields] OR "polyneuropathy"[All Fields]) OR "polyneuromyopathy"[All Fields] OR "polyneuromyopathies"[All Fields] OR ("Neuromuscular Diseases"[MeSH Terms] OR ("neuromuscular"[All Fields] AND "diseases"[All Fields]) OR "Neuromuscular Diseases"[All Fields] OR "neuromyopathies"[All Fields] OR "neuromyopathy"[All Fields]) OR ("Neuromuscular Diseases"[MeSH Terms] OR ("neuromuscular"[All Fields] AND "diseases"[All Fields]) OR "Neuromuscular Diseases"[All Fields] OR "neuromyopathies"[All Fields] OR "neuromyopathy"[All Fields])) OR ("ieee comput intell mag"[Journal] OR "clin invest med"[Journal] OR "cim"[All Fields] OR "CIP"[All Fields] OR "CIPM"[All Fields] OR "CIPNM"[All Fields] OR "ICUAW"[All Fields] OR "ICUAP"[All Fields]) OR "CINMA"[All Fields])
